# Modeling an Evolutionary Conserved Circadian *Cis*-Element

**DOI:** 10.1371/journal.pcbi.0040038

**Published:** 2008-02-15

**Authors:** Eric R Paquet, Guillaume Rey, Felix Naef

**Affiliations:** 1 Swiss Institute of Bioinformatics (SIB), Lausanne, Switzerland; 2 Swiss Institute of Experimental Cancer Research (ISREC), Ecole Polytechnique Fédérale de Lausanne (EPFL), Lausanne, Switzerland; Duke University, United States of America

## Abstract

Circadian oscillator networks rely on a transcriptional activator called CLOCK/CYCLE (CLK/CYC) in insects and CLOCK/BMAL1 or NPAS2/BMAL1 in mammals. Identifying the targets of this heterodimeric basic-helix-loop-helix (bHLH) transcription factor poses challenges and it has been difficult to decipher its specific sequence affinity beyond a canonical E-box motif, except perhaps for some flanking bases contributing weakly to the binding energy. Thus, no good computational model presently exists for predicting CLK/CYC, CLOCK/BMAL1, or NPAS2/BMAL1 targets. Here, we use a comparative genomics approach and first study the conservation properties of the best-known circadian enhancer: a 69-bp element upstream of the *Drosophila melanogaster period* gene. This fragment shows a signal involving the presence of two closely spaced E-box–like motifs, a configuration that we can also detect in the other four prominent CLK/CYC target genes in flies: *timeless, vrille, Pdp1,* and *cwo.* This allows for the training of a probabilistic sequence model that we test using functional genomics datasets. We find that the predicted sequences are overrepresented in promoters of genes induced in a recent study by a glucocorticoid receptor-CLK fusion protein. We then scanned the mouse genome with the fly model and found that many known CLOCK/BMAL1 targets harbor sequences matching our consensus. Moreover, the phase of predicted cyclers in liver agreed with known CLOCK/BMAL1 regulation. Taken together, we built a predictive model for CLK/CYC or CLOCK/BMAL1-bound *cis*-enhancers through the integration of comparative and functional genomics data. Finally, a deeper phylogenetic analysis reveals that the link between the CLOCK/BMAL1 complex and the circadian *cis*-element dates back to before insects and vertebrates diverged.

## Author Summary

Life on earth is subject to daily light/dark and temperature cycles that reflect the earth rotation about its own axis. Under such conditions, organisms ranging from bacteria to human have evolved molecularly geared circadian clocks that resonate with the environmental cycles. These clocks serve as internal timing devices to coordinate physiological and behavioral processes as diverse as detoxification, activity and rest cycles, or blood pressure. In insects and vertebrates, the clock circuitry uses interlocked negative feedback loops which are implemented by transcription factors, among which the heterodimeric activators CLOCK and CYCLE play a key role. The specific DNA elements recognized by this factor are known to involve E-box motifs, but the low information content of this sequence makes it a poor predictor of the targets of CLOCK/CYCLE on a genome-wide scale. Here, we use comparative genomics to build a more specific model for a CLOCK-controlled *cis*-element that extends the canonical E-boxes to a more complex dimeric element. We use functional data from *Drosophila* and mouse circadian experiments to test the validity and assess the performance of the model. Finally, we provide a phylogenetic analysis of the *cis*-elements across insect and vertebrates that emphasizes the ancient link between CLOCK/CYCLE and the modeled enhancer. These results indicate that comparative genomics provides powerful means to decipher the complexity of the circadian *cis*-regulatory code.

## Introduction

In flies and mammals, circadian timing is controlled via interlocked transcriptional feedback loops that rely on basic helix-loop-helix (bHLH), PAS domain transcription factors [1,2]. In both fly and mammalian systems an evolutionary conserved bHLH heterodimer acts as the central transcriptional activator. The pair is called CLOCK [3] and CYCLE [4] in *Drosophila*, while the mammalian orthologues are CLOCK [5] and BMAL1 [6]. In mammals the CLOCK paralog NPAS2 can substitute for CLOCK function in the suprachiasmatic nucleus [7,8]. Like most transcription regulators of the bHLH family members, DNA binding of the CLK/CYC or CLOCK/BMAL1 pairs has been shown to involve canonical CANNTG E-box sequences [9–11] both in flies and mammals [6,12]. However, the low information content of this motif does not provide a sufficient explanation for the specificity of gene induction by the CLOCK transcription factor, nor does it allow to build a model that can predict clock regulated transcripts on a genome-wide scale.

Both the possibility of informative nucleotides flanking the E-boxes or the possibility that a combination of closely spaced partner signals could contribute cooperatively to the specificity was considered in flies and mammals [13,14]. Either mechanism can in theory significantly increase binding affinity of CLK/CYC to DNA, e.g. an increase in total Δ*G*_0_ of 1 kcal/mol from one additional good hydrogen bond raises binding affinity by a factor of 5.

In *Drosophila*, the best-studied enhancer is that of the *period* (*per*) gene where a 69-bp fragment upstream of the transcription start site (TSS) drives circadian gene expression [9]. This enhancer depends on a canonical E-box, but it was also shown that its immediate 3' flank contributes to drive large amplitudes and tissue specific expression [15]. Interestingly, the fly enhancer can also be activated by the murine CLOCK/BMAL1 complex [6]. The next best studied enhancer is that of the *timeless* (*tim*) gene [11] which harbors closely spaced E and TER boxes, the latter being a variant of the consensus E-box which coincides with the mammalian E'-box [16]. In the mouse, well-studied CLOCK/BMAL1 elements include the *Per1* [6], *Per2* [17], *Avp* [14] and *Dbp* [18] genes. A study of the *Avp* promoter suggested that CLOCK/BMAL1 enhancers use a combination of a canonical E-box and a second more degenerate version thereof [14]. More recently a pyrimidine-rich 22 nucleotides sequence was found to cooperate with the core E-box in the *Avp* promoter [19]. So far, however, it was not possible to compile this information to build a predictive algorithm for CLK/CYC or CLOCK/BMAL1-activated enhancers.

Computational strategies for the optimal discovery of *cis*-elements from genomic sequence pose formidable algorithmic challenges [20]. Among the many ways to model transcription factor binding sites, position weight matrices (PWMs) reflect most closely the biophysics of protein-DNA interactions [21–23]. Recent algorithms that exploit phylogeny to infer PWMs apply probabilistic (Gibbs) sampling to evolutionary models [24–26], or implement expectation maximization to optimize scoring schemes that incorporate phylogeny [27–30]. Most of these methods allow for relatively simple model architectures, mostly single block motifs or symmetric structures [31]. Hidden Markov Models (HMMs) [32] and their phylogenetic extensions [33,34] are best suited for more complex model structures like the one we use. The phylogenetic HMMs currently focus on optimizing trees [33] rather than motif identification; the latter would require optimizing the state dependent equilibrium frequencies. However conventional HMMs, for which motif training is well established, can be supplemented with a weighting scheme approximating the phylogenetic dependencies [35,36], which is what we will use here.

Our analysis starts with the five known CLK/CYC targets among the clock genes in *Drosophila*, *per* [9,10,37]*, tim* [38]*, vrille (vri)* [39]*, Par-domain protein 1 (Pdp1)* [40], and *clockwork orange* (*cwo*) (formerly CG17100) [38,41,42]. Starting from the 69-bp enhancer in the *period* gene, we found a *cis*-element that is both common to all five genes and highly conserved among *Drosophila* species. This enhancer, which we validate using functional data, not only refines the core circadian E-box (E1), but also incorporates a flanking partner element (E2) that resembles the more degenerate E-box discussed above, and which is found at a very specific distance of the core E-box with an uncertainty of one nucleotide. While such structures are not implemented in common motif discovery programs, they are conveniently modeled with hidden Markov models (HMMs) [32]. We thus trained such an HMM model from the available fly sequences. Remarkably, the *Drosophila* model was able to predict many known mammalian CLOCK/BMAL1 targets without modification and with high specificity. A deeper phylogenetic analysis revealed the presence of the *cis*-element throughout insects and vertebrates. This shows that despite important differences in the organism's clock architectures, e.g., rhythmic mRNA accumulation of *Clock* in flies versus *Bmal1* in mammals, an ancient element in the circadian *cis*-regulatory code has been maintained since their common ancestor 500 million years ago.

## Results

### Evolutionary Conservation of CLK/CYC Enhancers in *Drosophila*

**Figure 1:**
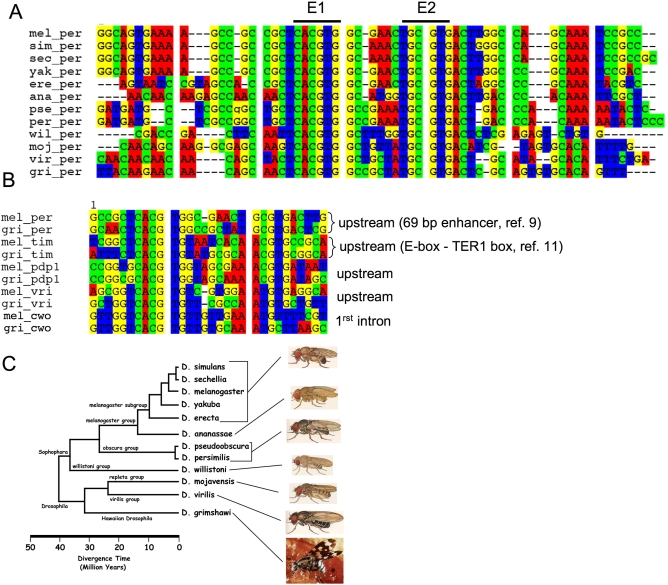
Period Enhancer and Related Sequences in Other CLK/CYC Targets (A) Alignment of the central part of the 69-bp enhancer in the Drosophila period gene in the 12 species. Locations of E1 and E2 boxes are indicated. (B) Similar sequences were found in promoters of the five known CLOCK/CYCLE targets: *period, timeless, vri, Pdp1,* and *cwo* (*CG17100*). Only the two most distant species are shown (D. melanogaster and D. grimshawi), but the elements are found across the full species tree. (C) Species tree of sequenced *Drosophila* (from http://rana.lbl.gov/drosophila).

The 69-bp enhancer upstream of the *per* promoter in D. melanogaster was discovered and dissected in great detail [9]. Using genome sequences from 12 *Drosophila* species [43,44], we searched for presence of this enhancer in this clade (Figure 1). Although not immediate to find (in current UCSC alignment the enhancer is absent in half of the species), we identified sequences in all species that show remarkable conservation in a ∼25 bp subfragment tightly collocated around the central canonical E-box motif (Figure 1A). The subfragment harbors a half E-box (GTG) located 9 bp to the right of the central E-box in the species close to D. melanogaster, and 10 bp for more remote clade members, e.g. D. grimshawi. Moreover the subfragment contains the 18 bp E-box [10] and the 3' flanking regions showing the strongest attenuation in activity upon deletion [15]. We then searched for similar flanking signals in the vicinity of other conserved E-boxes near promoters of validated CLK/CYC targets. We noticed that all five known target genes contain such dimeric signals that can be aligned with the *per* enhancer (Figure 1B), and also that this particular signal is conserved in all species considered.

### Deriving a Probabilistic Model from Five Known CLK/CYC Targets in *Drosophila*

**Figure 2:**
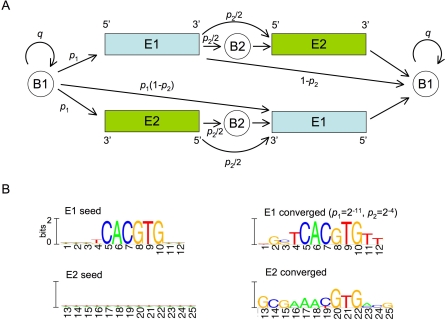
Probabilistic E1-E2 Model and Its Training (A) Structure of the circular E1-E2 hidden Markov model (HMM). All transition probabilities are indicated except when they are equal to 1, and *q* = 1 − 2*p_1_* − *p_1_*(1 − *p_2_*). The background states B1 and B2 have tied emission states; for the E1 (13 positions) and E2 boxes (12 positions) the emissions in the reverse strand model (lower part) are tied with those in the forward direction. (B) The model is initialized with the matrices in the left and converges to the matrices on the right. Transition probabilities are those used throughout: *p_1_* = 2^−11^, *p_2_* = 2^−4^. All models are given at http://circaclock.epfl.ch/Models. Matrices are displayed in information format (*I_a_* = max(0, *p_a_*log_2_(*p_a_*/*q_a_*)), where *p_a_* are the probabilities for letter *a* in a column and *q_a_* are the (genomic) background frequencies.

**Figure 3:**
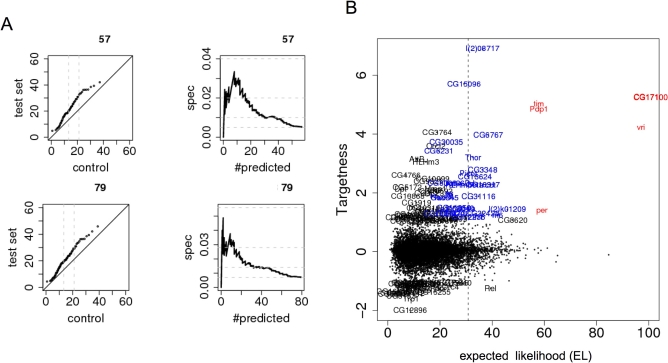
Promoters of Highly Induced Genes in the GR-CLOCK Are Enriched with High Scoring Hits from the E1-E2 Model (A) Left two diagrams: quantile–quantile plots show that expected likelihood (EL) scores of highly induced genes (positives) are shifted upward with respect to the control (negatives). Positives correspond to the 57 (top 0.5%; upper left diagram) or 79 (top 0.7%; lower left diagram) induced genes (ranked according to fold induction; see Methods), while the negative set consists of all remaining genes. Right two diagrams: Specificity versus number of predicted genes (sensitivity) in the group of 57 (upper right diagram) or 79 (lower right diagram). The horizontal lines represent expected specificity (lowest line), 2-fold, 4-fold, and 8-fold enrichment. Importantly, the five training genes are excluded from the set of positives in all panels. The increased specificities are highly significant: in the top row, *p* = 1.4 × 10^−7^ for 10 predicted positives (chi-squared test), *p* = 7.5 × 10^−6^ for 20, and *p* = 1.3 × 10^−3^ for 30 predicted positives. The top 30 positives are marked in blue in (B). (B) Scatter plot representing the targetness score (fold induction in log_2_ units; see Methods) in function of the expected log-likelihood score of the E1-E2 model in windows of ±2,500 bp around the TSSs. Genes in blue are the 30 genes (from the group of 57) with highest match to the sequence model.

To make this more systematic we focus on the vicinity of all conserved E-boxes that can be found around the TSSs of the circadian transcripts per-RA, tim-RA, Pdp1-RD, vri-RA, and cwo-RA. We used multiple alignments from the UCSC browser (http://genome.ucsc.edu/) and considered all islands of ± 30 bp around degenerate CANNGT sequences that were present at least in the subclade consisting of *D. melanogaster, D. yakuba, D. simulans, D. sechellia,* and *D. erecta* (in total about 660 nucleotides per gene for each species, available at http://circaclock.epfl.ch/training_seqs.fa). While conservation often extends to all 12 species, sub-optimal alignments required that we apply this milder criterion (cf. alignment of *per*, Figure S3A). A preliminary motif finding analysis of this restricted set of sequences based on the MEME algorithm [45] (using motifs length of 7) confirmed the presence of E-box-like dimers in these sequences (Figure S1). These were spaced with an accuracy of plus or minus one base pair as in the *per* enhancer (Figure 1A). To model this configuration we implement a HMM reflecting the dimer structure (Figure 2A), and train the emission probabilities from the example sequences using the Baum-Welsh algorithm [32]. The model is cyclic so that several instances of the motifs can occur per sequence, we also allow to by-pass E2 in the case that it would not be sufficiently supported by the training sequences. We seeded the model only with one E-box (Figure 2B, left) flanked by a weak T nucleotide to break the palindrome symmetry of the bare E-box, while the putative partner site (E2) is initialized with a fully uninformative model. Only the emissions are trained while the transition probabilities *p*_1_ from background to E1, and *p*_2_ form E1 to E2 are held fixed (Methods). These transitions tune the stringency of the E1 and E2 parts, and reflect the chemical potential of the regulators that would bind to the E1 and E2 boxes [23]. We varied *p*_1_ and *p*_2_ over a wide range and retained the combination that maximizes the enrichment of hits among genes that show induction by CLK in functional genomics assays (Figure 3). Importantly, despite the uninformative seed and large search space, converged models do reflect the right flank described above for a wide range of transitions, the combination retained (*p_1_*=2^−11^, *p_2_*=2^−4^) show a AACGTG right consensus. Apart from details in the emission probabilities, this model is quite stable for a range of *p*_1_ and *p*_2_ values (Figure S2).

Inspection of the converged model indicates that effectively 15 high scoring instances of E1 box were used, and 6 for the E2 box. The latter were from *vri* (2–3 instances), *per* (1–2), *tim* (1), *Pdp1* (1) and *cwo* (1). In these five genes it is noticeable that multiple E1-E2 copies are found, and that E1 also often occurs alone (Figure S3). For instance, the second conserved site in the *per* intron (Figure S3A) could provide an explanation for the promoterless *per* allele found to cycle in a restricted part of the nervous system [46]. Thus, the converged model is consistent with the attenuated CLK/CYC activation in mutated 69-bp enhancers with deletions that are immediately 3' of the right central E-box [15]. Furthermore the model captures the mammalian architecture in which a canonical and a fuzzier E-box are juxtaposed [14].

### Model Validation Using Functional Genomics Datasets

Training a model on five genes raises the question about its generalization to further putative CLK/CYC targets. To address this we used several microarray datasets that measure ‘CLK targetness' [38] (Methods) and assessed correlation with sequence match from our model. Windows of ±2500 bp around all annotated TSSs were scanned with our HMM model, in which the five training genes were found among the first 13 highest scores (Figure 3B).

Recently a glucocorticoid receptor-CLK fusion protein (GR-CLK) was used in S2 cells and cultured fly heads to induce CLK targets under cycloheximide treatment [38]. In this assay new protein synthesis is blocked to minimize indirect effects. Even though it is not formally excluded that the fusion protein could interfere with partner complexes, this experiment is best suited to test the specificity of the sequence model. We show that highly induced genes in the GR-CLK experiment are significantly enriched in high scoring hits from the sequence model, so that we can identify a set of ∼30 genes among the top 57 induced genes which show highly significant 2- to 6-fold enrichment in sequence specificity (Figure 3A and Table S1). Importantly, the five training genes are excluded from the set of positives in this analysis. When testing how much E2 contributes to the observed enrichment, we found that it contributes only marginally: it reduces specificity for low sensitivities and increases specificity at higher sensitivities (Figure S4A). Nonetheless, several of the highly induced genes in the GR-CLK experiment, e.g., *CG13624*, show presence of E1-E2. Moreover, these sites show highly increased conservation profiles specifically at the predicted locations including the E2 site (Figure S3F and S3G). Below we show that increased specificity from E2 is most important in mammals.

We also considered expression levels in *Clk^Jrk^* flies [47,48] since CLK/CYC targets are predicted to be down-regulated in this mutant. Moreover we tested cycling transcripts in light-dark (LD) and dark-dark (DD) conditions with phases that are compatible with known CLK/CYC targets, i.e., peak time accumulations in windows ZT6–20 (Methods). No signature of enriched E1-E2 motifs was detected in either the *Clk^Jrk^* or cycler datasets (Figure S4). This can be expected since both differential expression in *Clk^Jrk^* mutants, or rhythmic mRNA accumulation, also reflect indirect mechanisms downstream of the CLK/CYC transcription factor. We extensively searched whether other *p*_1_ and *p*_2_ parameters would detect enrichment without success. Consistently, we do not detect enrichment of the motif in mouse transcripts showing differential expression in a recent mRNA profiling of *Clock* mutants [49] (Figure S5). Similarly, in an early study of rhythmic transcript profiles in fly heads, we did not detect enrichment of consensus E-boxes in the vicinity of periodic transcripts [50].

Further annotating the list of 57 GR-CLK induced genes with the sequence score from the E1-E2 model, the 24-hour periodicity and phase of the transcripts in LD and DD, or with the differential regulation in *Clk^Jrk^* flies show that some genes qualify as CLK/CYC regulated genes according to several independent criteria (Table S1). Among those, the C2H2 zinc finger transcription factor *cbt, CG3348, CG11050, CG8008* are the most noticeable. From the purely genomic side, conserved E1-E2 sites are enriched in D. melanogaster when compared to permuted E1-E2 matrices (Figure S6A and S6B). From the likelihood scores of known examples, we estimate about one hundred genes to be potentially controlled by medium to high affinity E1-E2 sites (Figure S6C, gene lists in at http://circaclock.epfl.ch/fly_conserved_16.txt).

### The *Drosophila* E1-E2 Model Also Predicts Circadian Genes in the Mouse

**Figure 4:**
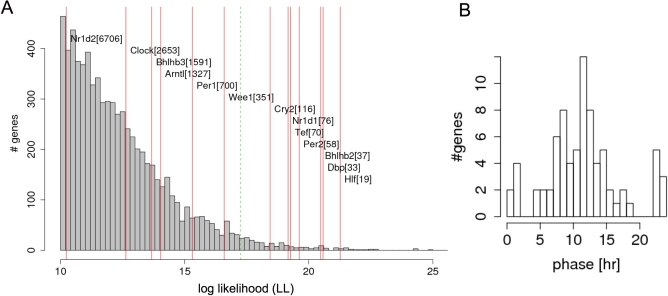
Genome-Wide Scan of the *Drosophila* E1-E2 Model onto the Mouse Genome (A) Genome-wide rankings of circadian mouse genes in function of their sequence likelihood score. The E1-E2 chained matrices are scanned along all mouse genes including ±2 kb flanking sequence (Methods). Only hits with average PhastCons scores above 0.5 are counted (in PhastCons 0 implies no conservation and 1 maximal conservation). The unconstrained result is shown in Figure S8. The name of the gene is always aligned to the right of its score line (in red), and its rank is indicated in brackets. A total of 7 of 13 expected circadian genes (Methods) are found above the 1% line (green dashed line), while we expected zero at this cutoff (*p* < 10^−12^). Notice Wee1 is just below the 1% line; E1-E2 is in the 3' region for this gene (Figure S7). Known circadian genes represented are *Cry1, Cry2, Per1, Per2, Per3, Dbp, Tef, Hlf, Wee1, Bhlhb2 (Dec1), Bhlhb3 (Dec2), Nr1d1 (RevErbα), Nr1d2 (RevErbβ), Arntl (Bmal1),* and *Clock.* The latter two are expressed in anti-phase with respect to known target and are mainly controlled by *Ror* orphan receptors and their repressors *RevErbα,β* [70]. Thus, *Clock* and *Bmal1* are not included in the test set. (B) Phase distribution of all conserved hits (as in [A]) with scores above 15 bits, and which show cycling in the liver in [54] (Fourier component F24 > 0.1; Methods).

Even though the model was derived from fly sequences, the core E-box shows similarities to the brain-specific in vitro measured NPAS2/BMAL1 binding consensus GGGTCACGTGT[TC]C[AC] (underlined bases are consistent with our model) [51]. Scanning the mouse genome with the full E1-E2 model taken straight from the flies revealed that many common circadian transcripts show instances of this signal that are highly conserved in mammals (Figure S7). Several of these genes also contain multiple instances of the motif, as in the flies. With few exceptions, sites are found in the vicinity of the core promoter (e.g., *Per2*, *Tef*) or in the introns (*Dbp*, *Cry2*, *RevErbα*). Given the much greater complexity of mammalian genomes as compared to insects, it comes as a great surprise that the fly model predicts known circadian genes in mouse with highly enriched specificity (Figure 4). Among the 13 common circadian genes used as a test set, we find 7 among the top 1% of predictions when we would expect none (*p* < 10^−12^, binomial distribution). In addition the restriction to sites that are highly conserved in mammals (measured using PhastCons [52]) increases the specificity (compare Figures 4 and S8). From the scores of known examples, we thus estimate in the order of hundred CLOCK/BMAL1 binding sites in mouse (Figure S6D). Finally, the two spacer lengths were about equally represented among the conserved hits with scores above 15 bits (given at http://circaclock.epfl.ch/bedFiles).

Importantly, while the E2 sequence played a marginal role in the specificity analysis of the GR-CLK data in flies, it plays a much more prominent role in mouse. For example, the *Dbp* site ranks only at position 804 and that of *Per2* at position 3021 when E2 is not used in the prediction (Figure S8, right); overall the 13 test genes are clearly shifted to the bulk of scores. The conservation pattern of many of these hits shows tight increase around the E1-E2 sequences (Figure S7), which further supports the functional role of the predicted loci. Moreover, several of these predictions coincide with known CLOCK/BMAL1 functional circadian enhancers, e.g., those in the *Per1* [6], *Per2* [17] or *Dbp* [18] genes. As with the Drosophila *Clk^Jrk^* data, putative CLOCK/BMAL1-induced genes identified from a *Clock* mutant array experiments in mice [49] did not show enriched E1-E2 boxes presumably due to indirect effects, except perhaps for a weak tendency in the liver (Figure S5). Consistent with our model, recent circadian band shift assays with mouse liver extracts indicate that a sequence closely related to the E1-E2 site is able to shift the CLOCK/BMAL1 complex more specifically than single E-boxes [53]. Finally, the phase distribution among the conserved hits that cycle in liver [54] shows a clear phase preference around ZT12, as expected for CLOCK/BMAL1 targets (Figure 4B).

### Deep Evolutionary Conservation of the E1-E2 Motif

**Figure 5:**
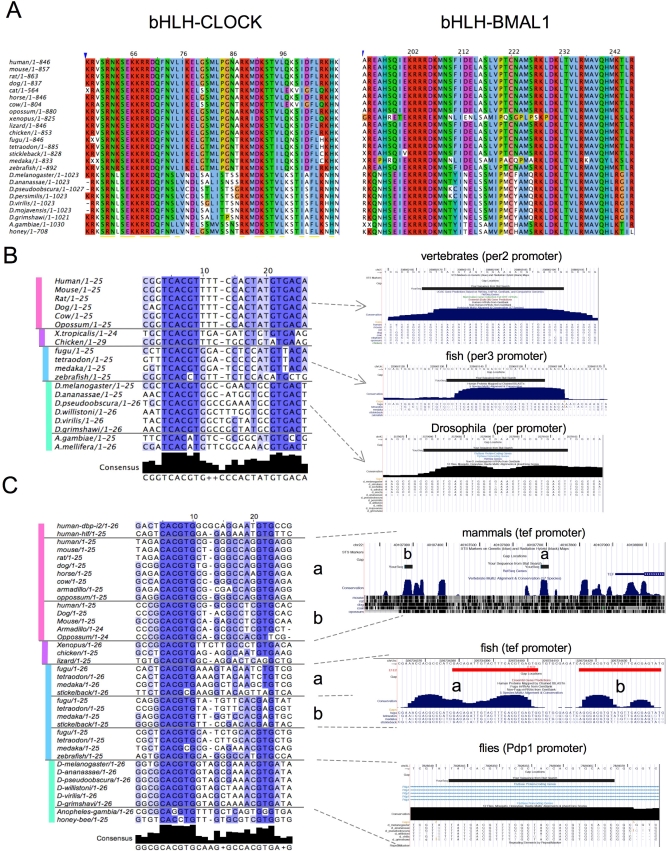
Deep Evolutionary Conservation of the CLOCK/BMAL1 Proteins and Their Target Sites (A) Partial sequence alignment of the bHLH protein–DNA interaction domain in the CLOCK and BMAL1 proteins shows high conservation throughout vertebrates and insects. (B) Left: E1-E2 motifs in Period genes; for space reasons too redundant species (e.g., the apes) are not shown. Notice the similarity in E2 between fish and mammals. Right (top): the murine *Per2* enhancer located near the TSS [17] can be aligned up to chicken. Right (middle): conserved E1-E2 enhancer upstream of *Per3* gene (2.6 kbp upstream of the start codon, the TSS is unannotated). Right (bottom): the *period* enhancer in flies (idem Figure S3A), positioned at −530 in D. melanogaster. As explained in the text, the more distant flies are missing from the MultiZ alignment, even though the enhancer is present (Figure 1A). (C) Left: E1-E2 motifs in *Tef* genes. Sites in the human paralogues *Dbp* and *Hlf* are shown in the first two lines. Two sites are shown for the mammals and three for the fish. Right (top): the 5' end of the *Tef* mRNA is shown with two conserved E1-E2 sites (see Figure S7G) located at −200 (a) and −600 (b) bp. Right (middle): two closely spaced E1-E2 sites in the *Tef* promoter at −500 bp of the putative start site. Right (bottom): the enhancer in the *Pdp1* gene (Figures 1B and S3D) at −2.2 kbp of the Pdp1-RD transcript.

We first provide a phylogenic analysis of the activators CLOCK/BMAL1 binding E1 in mammals, birds, frogs, fishes, flies, mosquito and honey bee. Beyond these species, notably in the nematodes, no orthologues can be found. Both CLOCK and BMAL1 harbor two conserved PAS domains, in addition to the preserved DNA binding bHLH domain (Figure 5A; full-length protein alignments are given at http://circaclock.epfl.ch/jarFiles), whose conservation exceeds by far the bHLH consensus motif [55,56]. As the complex is expected to bind the E1 site, its conservation is consistent with the high information content (11.0 bits) of the E1 motif.

To track the presence of the E1-E2 motif in a broader set of species, we consider two gene families among the best conserved circadian CLK/CYC or CLOCK/BMAL1 targets. First, the *Period* genes are primary targets of CLOCK/BMAL1 whose genes products function as repressors of CLOCK/BMAL1, hence closing a negative feedback loop at the core of the circadian oscillator. While flies have a single *period* gene, vertebrates have multiple copies, e.g., three in mammals. The presence of E1-E2 signals near promoters of *period* genes generalizes beyond flies and mammals to a broad set of species including birds, frogs, fishes, flies, mosquito and honey bee (Figure 5B). While the mammalian site is at the TSS and that of fly is around −500 bp, the fish promoter is unannotated and the site is at 2.6 kbp upstream of the annotated PER3 protein. Interestingly the mammalian E2 motif shares many similar bases with the fish. Even though nematodes have a putative *period* homologue (*lin-42*), we could not detect presence a proximal E1-E2 in C. elegans and C. briggsae, which is both consistent with the absence of CLOCK/BMAL1 and the still uncertain existence of circadian rhythms in nematodes [57].

Second, the PAR-domain basic leucine zipper (PAR bZip) transcription factors *Tef/Hlf/Dbp* (mouse) are homologues of the fly circadian gene *Pdp1* (PAR domain protein 1) and are prominent clock output genes directly regulated by E-box motifs [12,18]. Their function is to mediate rhythmic physiology in organs such as the liver and kidney, where they induce, e.g., the cytochrome P450 enzymes [58]. Among the three murine paralogues, *Tef* is the most ancient representative with putative orthologues in most vertebrates and insects. In few species, e.g., in zebrafish and Xenopus tropicalis, full-length mRNA are available for *Tef*, elsewhere we relied on annotations inferred from a combination of ESTs and proteins (from other species) to genome alignments provided in the UCSC web browser. We could find E1-E2 elements in the vicinity of the *Tef* promoter in most of the vertebrates and insects, some harboring several copies (Figure 5C). Interestingly, the locations of the instances of the E1-E2 motif shows a typical conservation structure (in the PhastCons scores) in subgroups where non-coding sequences can be multiply aligned, i.e., the mammals, the fishes, and the flies.

Even if the exact position of the TSS is poorly documented in many of these species, we find that more than 85% of the shown sequences for both the *Period* and *Tef* genes occur within 1.5 kbp of an annotated start. Furthermore, 75% (respectively 25%) of the likelihood scores are above 15.1 bits (respectively 19.5 bits) and the median score is at 17.1 bits. Using background statistics for the E1-E2 likelihood score computed as in [23] (Figure S9), we estimate that the probability per position to find a motif having a likelihood score greater than 17 bits is 5 × 10^−7^, or 2 × 10^−6^ for scores of 15 bits. Assuming independent positions, we estimate that the probability *p* to find conserved hits (PhastCons > 0.5) in regions of 1.5 kbp around the mammalian, fish and insect promoters is *p* = 2 × 10^−9^ for 17 bits hits and *p* = 10^−7^ for 15 bits. Here we used that the genomic fraction of conserved sites (PhastCons > 0.5) is 10% in mammals (UCSC mm8 assembly, PhastCons score based on 18 species), 23% in fish (fr2 assembly, 4 species), and 40% flies (dm3, 15 species). This simple calculation thus suggests that the conserved configurations found for the *Period* and *Tef* genes are highly unlikely due to chance.

## Discussion

Even though novel post-transcriptional mechanisms regulating the circadian clockworks are regularly uncovered [59], transcriptional control remains an essential ingredient of molecular clocks that is particularly relevant for relaying circadian output functions [2]. Output genes can be induced by the transcription factors of the core oscillator, or via tissue specific effectors such as *Dbp*, *Hlf* and *Tef* in mouse, which are themselves direct CLOCK/BMAL1 targets [58]. This layered design complicates the interpretation of experiments such as mRNA steady state time courses, particularly if one is interested in deciphering new direct targets of the core regulators. This task can be greatly facilitated using functional experiments like the glucocorticoid-CLK fusion experiments, which have improved specificity compared with the profiling of mutants, and accurate models for the *cis*-regulatory sequences bound by the regulators. Presently the mechanisms that facilitate the recruitment to DNA and subsequent trans-activating activity of the main circadian regulator CLK/CYC or CLOCK/BMAL1 are not fully understood. Likely though, this situation will evolve rapidly, helped by approaches such as large-scale chromatin immuno-precipitation analyses or comparative genomics. We used the latter to derive a probabilistic model for CLK/CYC-regulated circadian enhancers consisting of two partner signals, E1 and E2, linked by a spacer that can tolerate a variability of one nucleotide. E1 has an E-box core flanked by informative T's (or A on the reverse strand), while the second half is more degenerate and resembles previously reported TER boxes [11] or E' boxes [16]. The close proximity of the two sites suggests a cooperative binding of two partner complexes, one of which is the CLK/CYC heterodimer, while the second possibly identical factor needs to be identified.

To validate the predictive power of the model in *Drosophila,* we analyzed a recent study in which a GR-CLK fusion was used to induce CLK/CYC targets in S2 cells. We found an unusual number of high sequence scores among the highest induced genes, even though the E2 part did not contribute a large improvement in this case. This could reflect two scenarios: either the fusion protein interferes with a putative E2 binding complex, or it could simply be that the list of highest affinity CLK/CYC targets does not extend much beyond the list of known five, even though we identified several strong candidates that harbor the expected *cis*-element (Figure S3 and Table S1). Consistent with the first functional study of the period enhancer [9] we find no preferential orientation of the E1-E2 elements. Anecdotally, it is interesting that the double E1-E2 site around −2.5 kb in the *vrille* promoter (Figure S3C) is located on a fragment that is inverted in D. grimshawi only (Figure S10).

Having built the model from *Drosophila* sequences only, it was quite remarkable that the unchanged E1-E2 model identified high scoring hits in the majority of known CLOCK/BMAL1 targets in mouse. Among genes with putative E1-E2 elements, many instances of the motif are highly conserved, and the conservation patterns are often concentrated just on top of the identified elements while rapidly decreasing outside of it. Unlike in flies, the E2 element appears to be a determinant for specificity in mouse. Given that tissue-specific expression analyses [60,61] revealed largely non-overlapping circadian regulation programs, it is not excluded that future analyses will reveal enhancer elements permitting tissue specific predictions. We showed that our model predicted peak expression phases in mouse liver that were preferentially centered around ZT12 (Figure 4B), which is consistent with an induction by CLOCK/BMAL1. It might be possible to find subclasses in the E1-E2 model that drive expression with more specific phases, e.g., by modifying the binding affinity of the E2 element. There should nevertheless be limits to this undertaking as mRNA accumulation is also influenced by processes downstream of transcription. Noticeably, many of our predicted CLOCK/BMAL1 targets show non-cycling steady state mRNA abundances, at least when assessed in liver [54]. It is likely that some will cycle in other tissues, however, long mRNA half-lives can easily mask rhythmic transcription rates as has been reported for the albumin gene [62].

In conclusion we built a probabilistic sequence model, termed E1-E2, that predicts enhancers driven by the bHLH proteins CLK/CYC in insects and CLOCK/BMAL1 in mammals. This model not only refines the circadian E-box beyond its core nucleotides but also emphasizes the role of a flanking partner motif that may involve binding of a novel co-regulator complex. A deeper phylogenetic analysis showed that conserved instances of E1-E2 are found both in promoters of core circadian clock genes, and in genes mediating circadian output. E1-E2 seems to occur in vertebrates and insects but not in nematodes. This is perhaps not surprising as the existence of circadian behavior in nematodes is still controversial [57]. Absence of E1-E2 could also reflect the Coelomata hypothesis that groups arthropodes with chordates in a monophyletic clade [63]. In this perspective our findings would suggest that the CLOCK/BMAL1 based oscillator evolved after the nematodes separated from a common ancestor. Alternatively, the nematodes could have lost some oscillator components as a result of their live style in the soil, which largely shields them from daily light cues. Our report is not the first example of an ancient linkage between bHLH regulators and companion *cis*-elements. An even deeper conservation of a *cis*-regulatory element has been reported in proneural genes controlled by bHLH factors of the Hes family [64]. Several reasons, e.g., the necessity to maintain highly stable key developmental programs, were proposed to explain such unusually high conservation. Here, it is interesting that the BMAL1 protein, unlike genes in the *Period* or *Crytochromes* families, stands out as the only circadian component in the murine clock with no functionally redundant paralogues. The high degree of conservation in its target sites is thus consistent with the unique function of BMAL1 (CYC) as the master activator in the circadian network. We surely expect that comparative genomics combined with functional datasets will allow further dissecting the circadian and other *cis*-regulatory codes.

## Methods

### *Drosophila* sequence data.

MultiZ [65] Multiple alignments were downloaded from the UCSC table browser (Multiple alignments of 14 insects with D. melanogaster, dm3, April 2006, but we restricted these to *Drosophila* species). We used the Drosophila melanogaster genome and annotations version r5.1 to analyze windows of ±2,500 bases around all annotated transcripts. These sequences were used to identify flanking sequences around conserved CANNGT motifs in the five training genes; for the *period* gene we added the 69-bp enhancer from the species missed in the multiple alignment (Figure 1A and Figure S3A).

*Model training.* The sequences used for the model training are given at http://circaclock.epfl.ch/training_seqs.fa. We implemented a standard Baum-Welsh optimization in which each sequence is independent (no explicit use of the multiple alignments is made). We took into account phylogenetic relationships by attributing a geometric weighting reminiscent of [35] reflecting the *Drosophila* species tree (Figure 1C): droGri2: weight = 1/8, dp4: 1/8, droYak2: 1/16, droEre2: 1/16, droPer1: 1/8, droWil1: 1/4, droSim1: 1/32, dm3: 1/16, droAna3: 1/8, droSec1: 1/32, droMoj3: 1/16, droVir3: 1/16. Thus each gene is counted as one and we used fixed pseudo-count of 0.3 for each nucleotide. Species identifiers are those used in the UCSC alignments. Training is done on both strands simultaneously with tied (reverse complemented) emission probabilities using a custom HMM implementation following [32].

### Genome scans in *Drosophila*.

We scanned (decoded) windows of ±2,500 bp for all annotated transcripts (r5.1) with the cyclic E1-E2 model. The converged HMM model is provided at http://circaclock.epfl.ch/Models/M_11_4_0.3_3_2_13_0_1.mod, while the seed model is http://circaclock.epfl.ch/Models/seed.M_11_4_3_2_13_0_1.mod.

We used posterior decoding to compute the posterior state probabilities *P_si_* for state *s* at position *i* (Figure S3), and the expected likelihood (EL) for a sequence is computed as 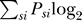 (*e_s_* (*O_i_*)) minus the likelihood of the background (Figure 3). Here, *e_s_*(*O_i_*) is the (emission) probability to observe nucleotide *O_i_* at position *i* in the state *s*. In the case of multiple transcripts, the highest score was used as the gene score. Correspondence between Affymetrix oligos and genes was done with the Annotations provided at NetAffx.com for the DrosGenome1 and Drosophila_2 arrays (July 2007 versions).

### Genome scans in mouse.

To scan the full mm8 mouse genome (from the UCSC genome browser) we extracted the two weight matrices from the *Drosophila* HMM (given at http://circaclock.epfl.ch/Models/M_11_4_0.3_3_2_13_0_1.p1.mat and http://circaclock.epfl.ch/Models/M_11_4_0.3_3_2_13_0_1.p2.mat), and computed the standard likelihood (LL) 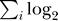 (*w_i_*(*O_i_*)/*b*(*O_i_*)) for the chained matrices at each genomic position. Here *w_i_*(*O_i_*) is the probability to observe nucleotide *O_i_* at position *i* and *b*(*O_i_*) is the background probability for nucleotide *O_i_*. As in flies we allow for a zero or one nucleotide spacer and consider the maximum of the two scores. We used a single nucleotide background (0-th order) with 29% of A and T's, and 21% of C or G's. To filter for conservation (Figures 4 and S8), we average PhastCons scores [52] (from alignments with 17 vertebrates, UCSC genome browser) at the positions of the hit (25 or 26 bases depending on spacer). Hits are mapped to genes when they occur in windows of ±2 kb of the transcription units from the affyMOE430 table at UCSC. The latter was used for easy comparison with expression data. A set of 15 known circadian genes was used to test the specificity of prediction in mouse: *Cry1, Cry2, Per1, Per2, Per3, Dbp, Tef, Hlf, Wee1, Bhlhb2 (Dec1), Bhlhb3 (Dec2), Nr1d1 (RevErbα), Nr1d2 (RevErbβ), Bmal1 (Arntl),* and *Clock*, of which the latter two are not expected to be self-induced.

### Array datasets.

Two *Clk^Jrk^* mutant time series of 12 time points each [47,48] were used to quantify differential regulation induced by the mutation, we applied a one-sample t-test to the 24 merged log_2_-expression ratios at each time point. GR-CLK induction data was from [38]; replicated conditions were averaged and the fold induction between stimulated and un-stimulated cells was computed separately for the S2 cells and the cultured fly heads. The two were then summed to make a single score for each gene. The obtained rankings correlate tightly with the original analysis. DD and LD cycling scores (amplitude and phases) were compiled from a comprehensive collection of previously described time courses [66] using established methods [67]. Information regarding genes induced or repressed in *Clock* mutant mice is taken from [49]. Mouse liver data used for Figure 3B is from [54], rhythmicity was assessed via the 24 hour Fourier component (F24) as in [67].

### Phylogenetic analysis.

The protein sequences for the CLOCK and BMAL1 homologues of insects and vertebrates, was taken from NCBI when available, and if not, we used the tBlastn table and the predicted protein from the UCSC database. The protein alignments were produced using ClustalW [68] and visualized using Jalview [69]. To identify instances of the E1-E2 motifs in the *Period* and *Tef* promoters, we used the UCSC browser to find the genomic regions around *bona fide* (when supported by full length mRNA in the specie) or putative (inferred from aligning mRNA, ESTs or protein from other species) homologues of these genes. We then scanned these sequences for instances of E1-E2 and the highest scoring instances near putative promoters were retained.

### Website.

Additional data and model files are given at http://circaclock.epfl.ch. Genes used for Figure 4B are listed in the file http://circaclock.epfl.ch/cyclers_mouse_fig4B.txt. Predictions (.bed file format) for flies and mouse can be uploaded to the UCSC Genome browser as custom tracks.

## Supporting Information

Figure S1 MEME Analysis of Sequences Surrounding Conserved CACGTG in Five Circadian Genes in Flies (1.7 MB PDF)

Figure S2 Models Obtained with Different Transition Probabilities *p1* and *p2*
(391 KB PDF)

Figure S3 Positions of E1 and E2 sites Around TSSs of Circadian Genes in Flies (4.5 MB PDF)

Figure S4 In *Drosophila,* E1-E2 Signal Is Not Enriched in Cyclic Genes or Genes That Are Downregulated in *Clk^Jrk^* Flies (1.0 MB PDF)

Figure S5 E1-E2 Enhancers in Δ19 *Clock* Mutants (262 KB PDF)

Figure S6 Estimated Number of E1-E2 Sites in Flies and Mice (527 KB PDF)

Figure S7 Position of E1-E2 Enhancers in Circadian Mouse Genes (1.3 MB PDF)

Figure S8 Sequence Conservation and E2 Improve the Specificity of E1-E2 Sites in Mouse (388 KB PDF)

Figure S9 Background Distribution for the Likelihood Score (163 KB PDF)

Figure S10 Sequence Inversion in the *D. Grimshavi vrille* Promoter (387 KB PDF)

Table S1 Top 57 GR-CLK–Induced Genes (77 KB PDF)

## Abbreviations

bp: base pair(s)
bHLH: basic helix-loop-helix
DD: dark-dark
EL: expected log-likelihood
GR-CLK: glucocorticoid receptor-CLOCK fusion
HMM: hidden Markov model
LD: light-dark
LL: log-likelihood
PWM: position weight matrix
TSS: transcription start site
