# Supplementary material for: Modeling an Evolutionary Conserved Circadian Cis-Element
Source: PLoS Comput Biol. 2008 Feb 15;4(2):e38. doi: 10.1371/journal.pcbi.0040038 (PMC2242825; doi:10.1371/journal.pcbi.0040038)
Supplement: Supplementary file 1 [file pcbi.0040038.sg001.pdf]

Supplementary material to E. Paquet, G. Rey, and F. Naef (PLoS Computational Biology).

Fig. S1

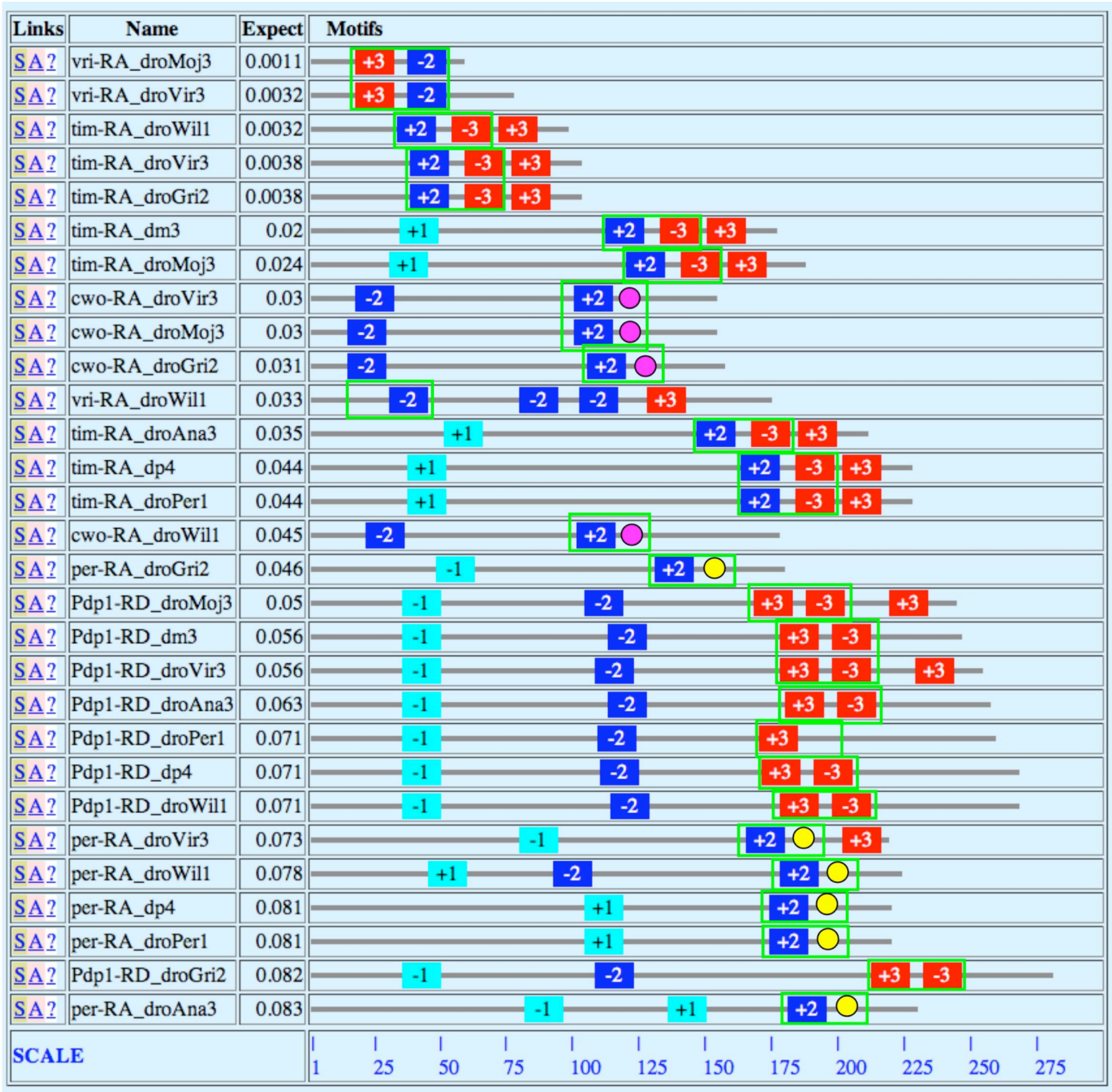

Figure S1. MEME analysis of sequences surrounding conserved CACGTG in five circadian genes in fly.

Motif analysis for sequences consisting of conserved CACGTG motifs (present in the UCSC multiple alignments in at least in the subclade consisting of *D. melanogaster, D. yakuba, D. simulans, D. sechellia and D. erecta*) plus their 30 bp flanks on each side. To avoid overcrowding with redundant species we hide the *D. yakuba, D. simulans, D. sechellia and D. erecta* sequences. The MEME program was run with the command 'meme -dna -mod zoops -nmotifs 3 -w 7 –revcomp', The output shown is from the companion program MAST with command 'mast -ev 0.1 -mt 0.0005'. The motifs indicated by boxes correspond to consensus ACACGTG (1), CACGTGT (2), CACGTTT (3); green rectangles indicate the E1 and E2 partner sites. The yellow circle indicate the TGCGTG variant in the *per* sequences, and the purple variant TTATGTG in that in *cwo* sequences. This analysis indicates the putative role of the double E-box like structure and motivates the double site structure that we train more systematically with the HMM approach.
