# Supplementary material for: Modeling an Evolutionary Conserved Circadian Cis-Element
Source: PLoS Comput Biol. 2008 Feb 15;4(2):e38. doi: 10.1371/journal.pcbi.0040038 (PMC2242825; doi:10.1371/journal.pcbi.0040038)
Supplement: Supplementary file 2 [file pcbi.0040038.sg002.pdf]

Supplementary material to E. Paquet, G. Rey, and F. Naef (PLoS Computational Biology).

Fig. S2

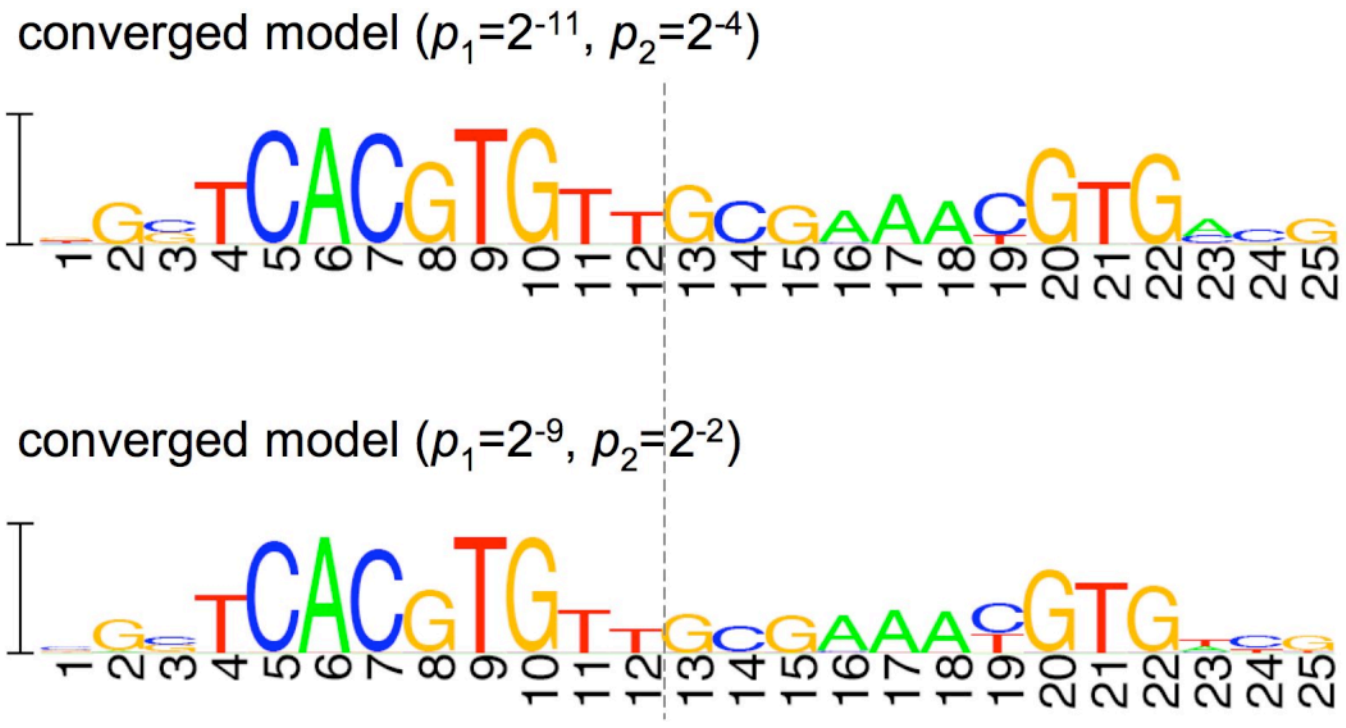

Figure S2. Models obtained with different transitions probabilities p_1 and p_2.

Top: model from Fig. 2A ($p_1=2^{-11}$, $p_2=2^{-4}$). The converged model uses approximately 15 high scoring instances of the E1 box and 6 for the E2 box in the training sequences (Methods). Bottom: ($p_1=2^{-9}$, $p_2=2^{-2}$), here the converged model uses 24 high scoring instances of E1 box and 10 for the E2 box. Both models are given at http://circaclock.epfl.ch/Models. Dashed line indicates the separation between E1 and E2.
