# Supplementary material for: Modeling an Evolutionary Conserved Circadian Cis-Element
Source: PLoS Comput Biol. 2008 Feb 15;4(2):e38. doi: 10.1371/journal.pcbi.0040038 (PMC2242825; doi:10.1371/journal.pcbi.0040038)
Supplement: Supplementary file 3 [file pcbi.0040038.sg003.pdf]

Supplementary material to E. Paquet, G. Rey, and F. Naef (PLoS Computational Biology).

Fig. S3A

per-RA

ref. [1], below

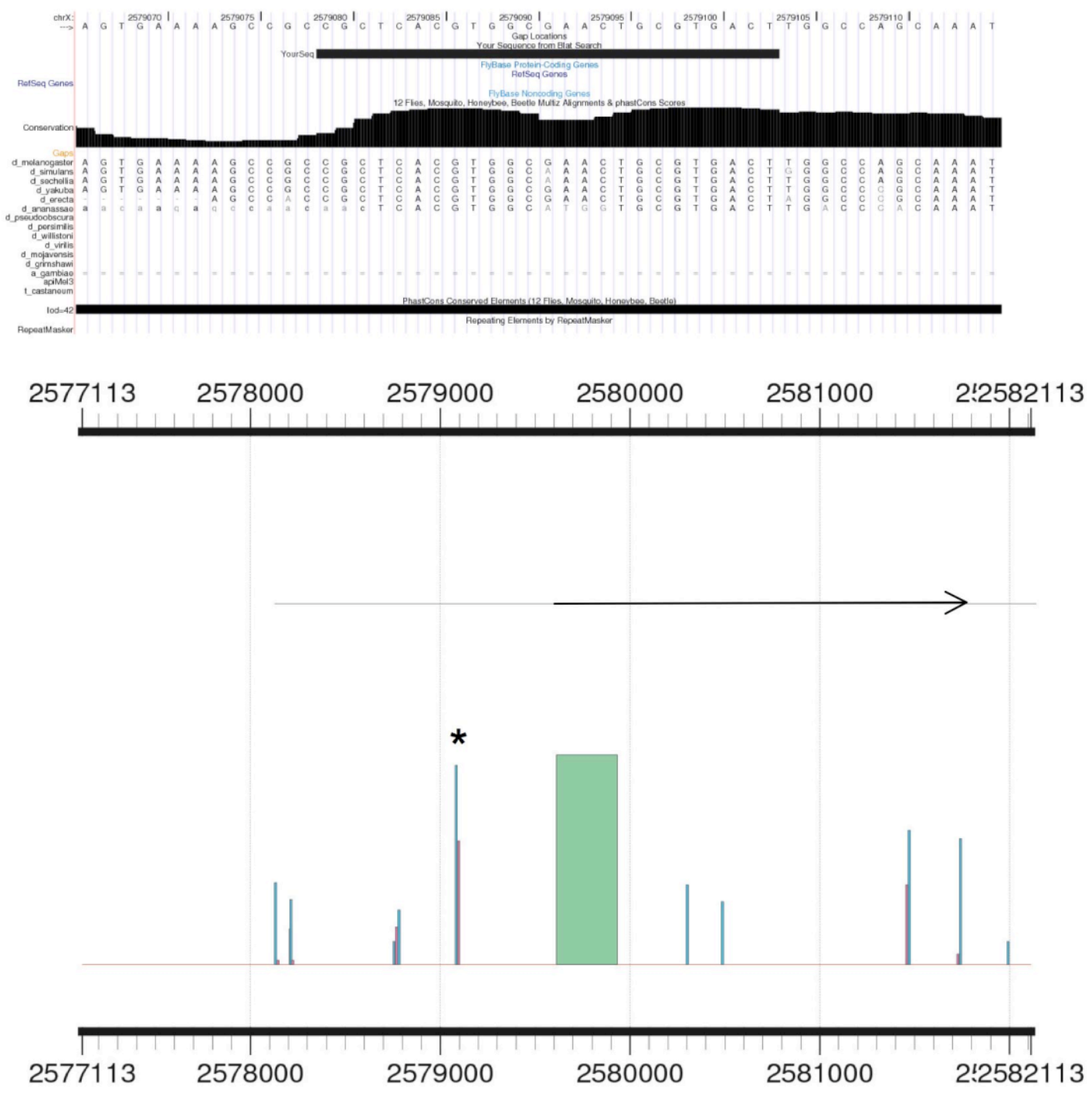

Fig. S3B

tim-RA

ref. [2], below

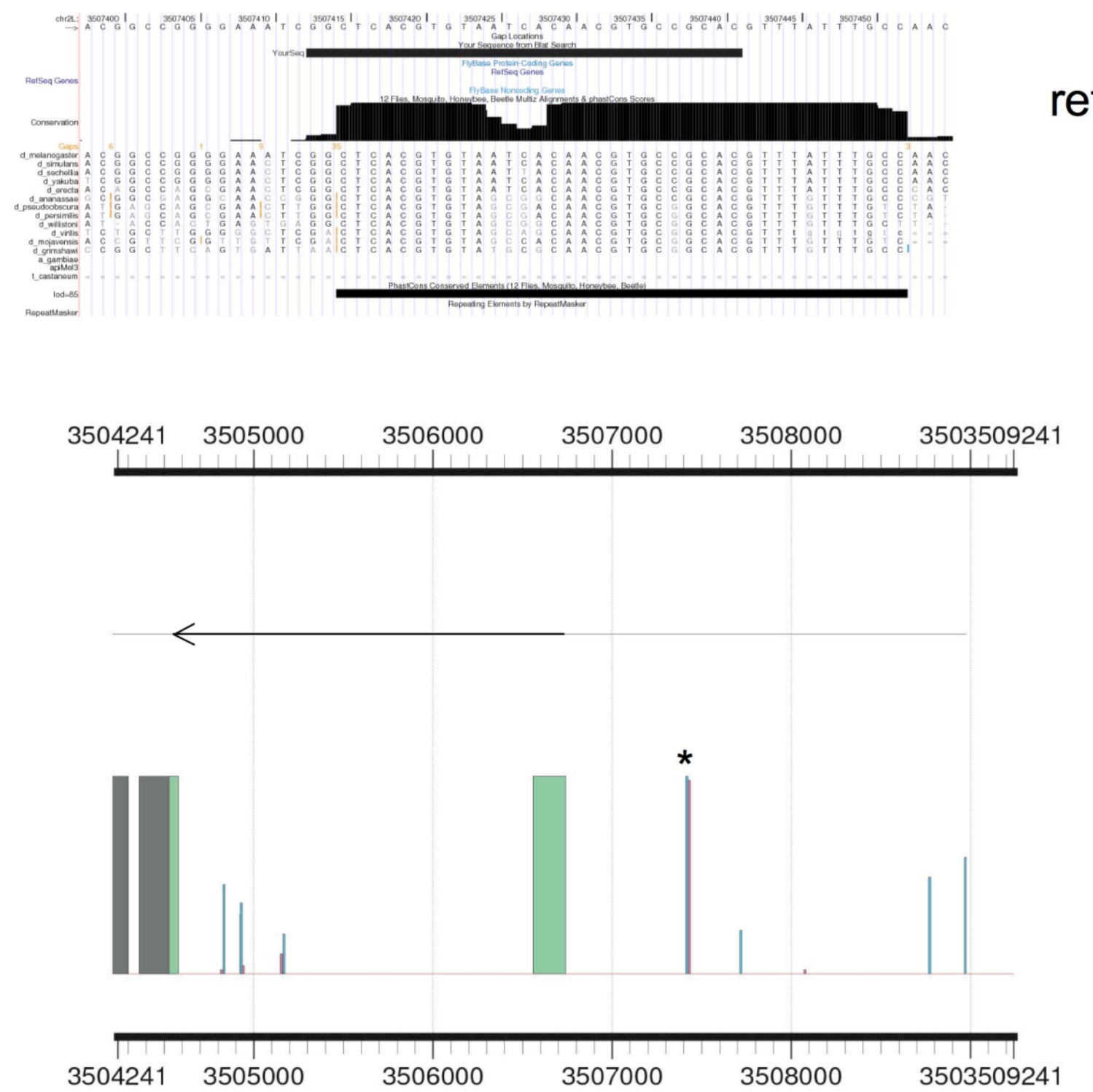

Fig. S3C

vri-RA

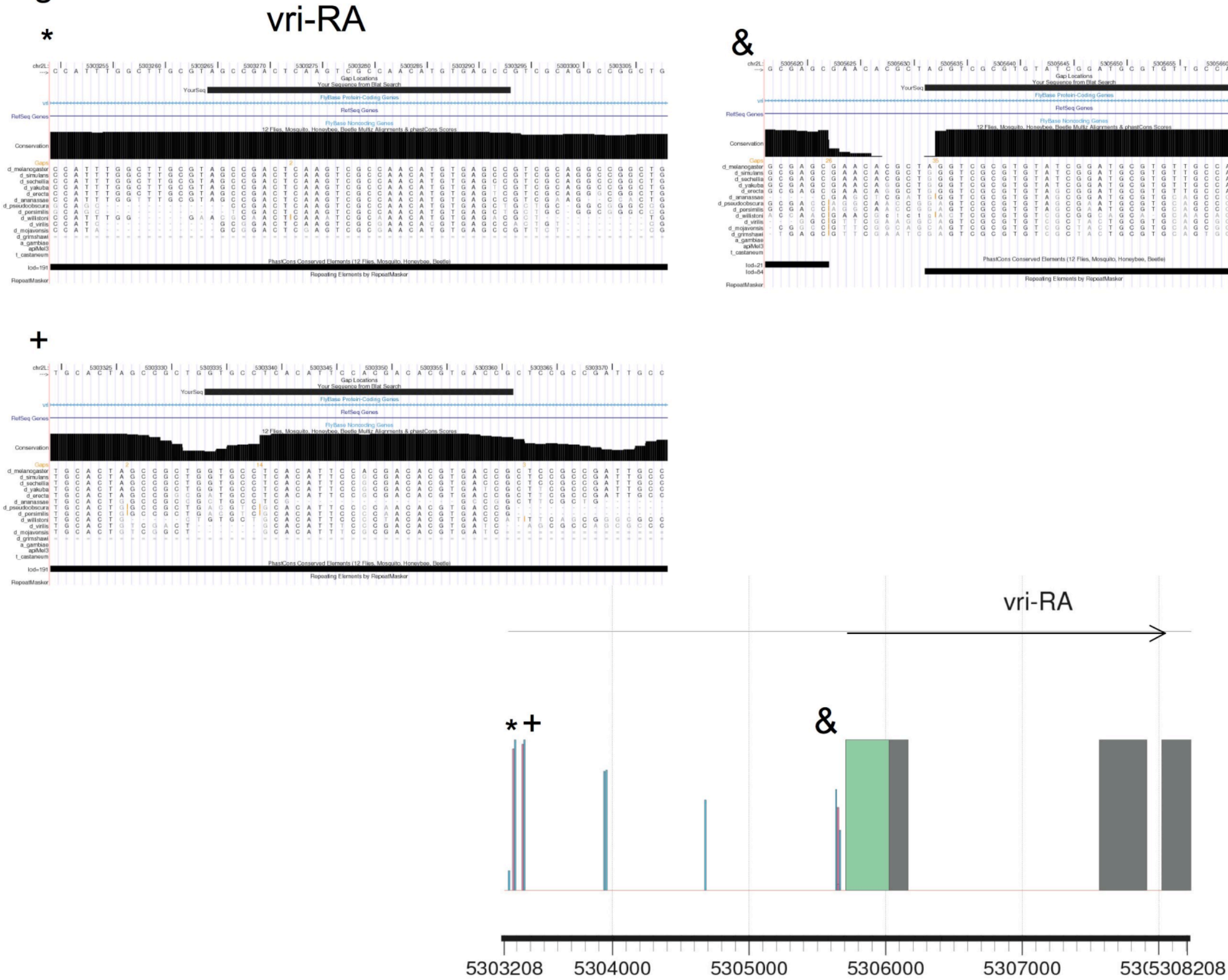

Fig. S3D

Pdp1-RD

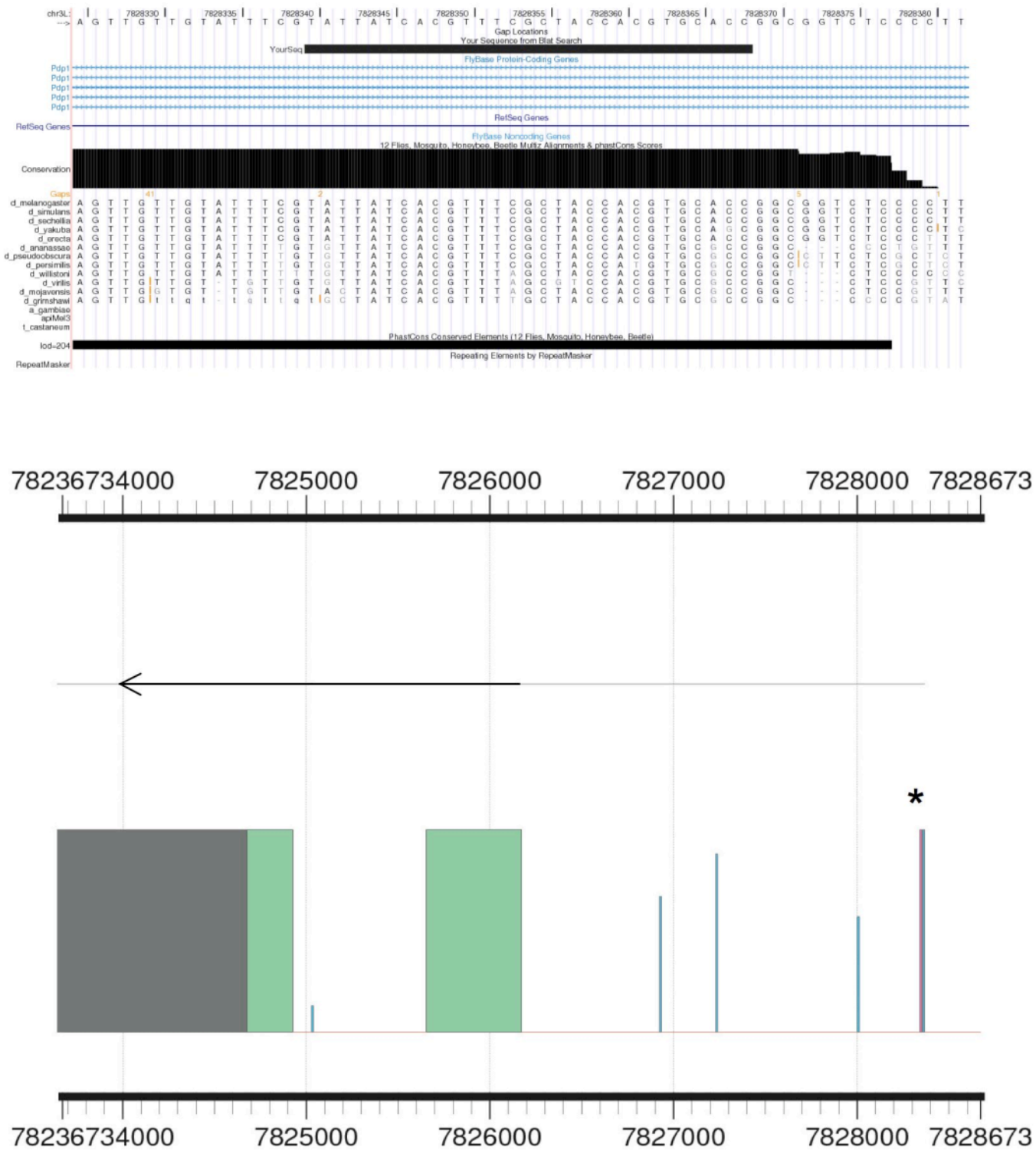

Fig. S3E CG17100-RA (cwo)

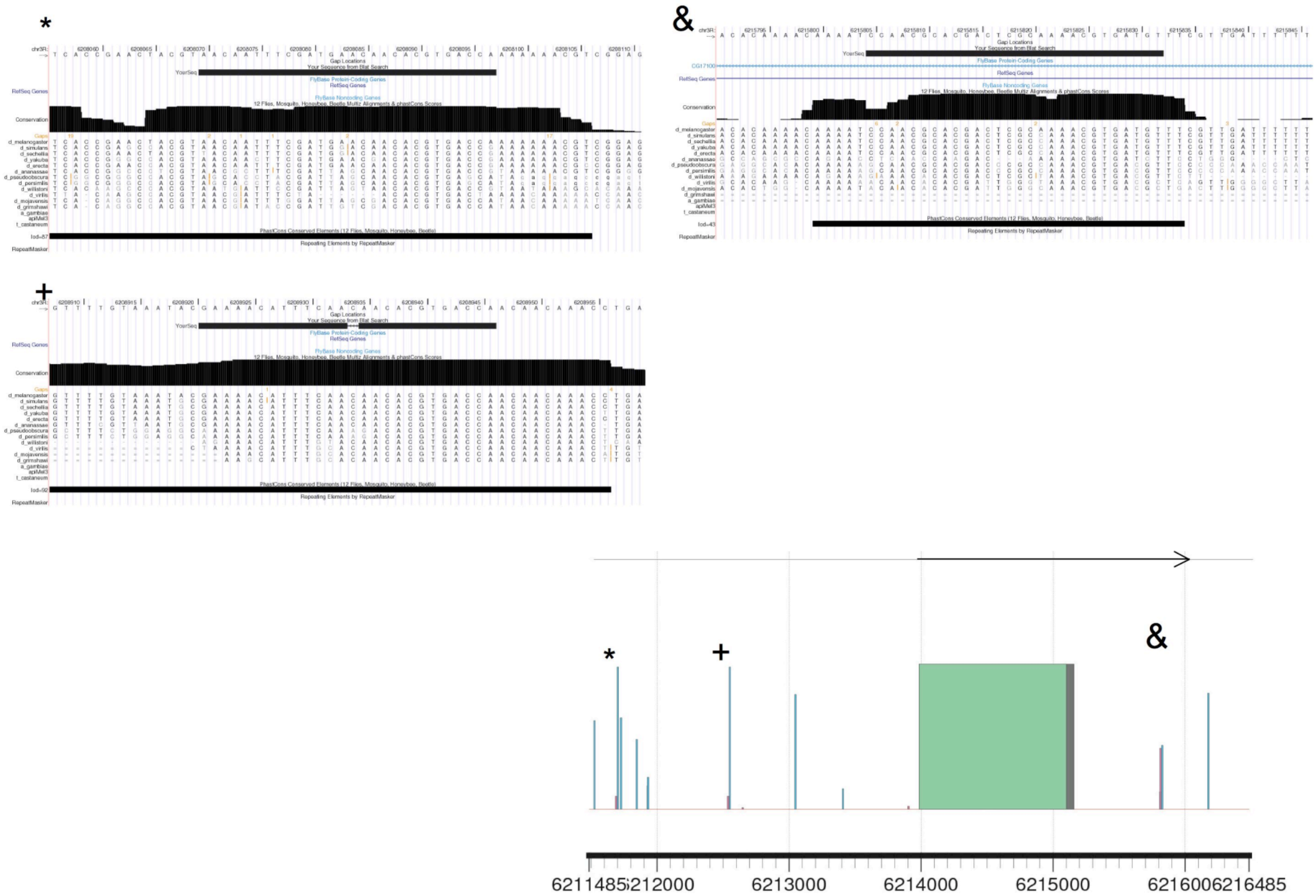

Fig. S3F CG13624-RA

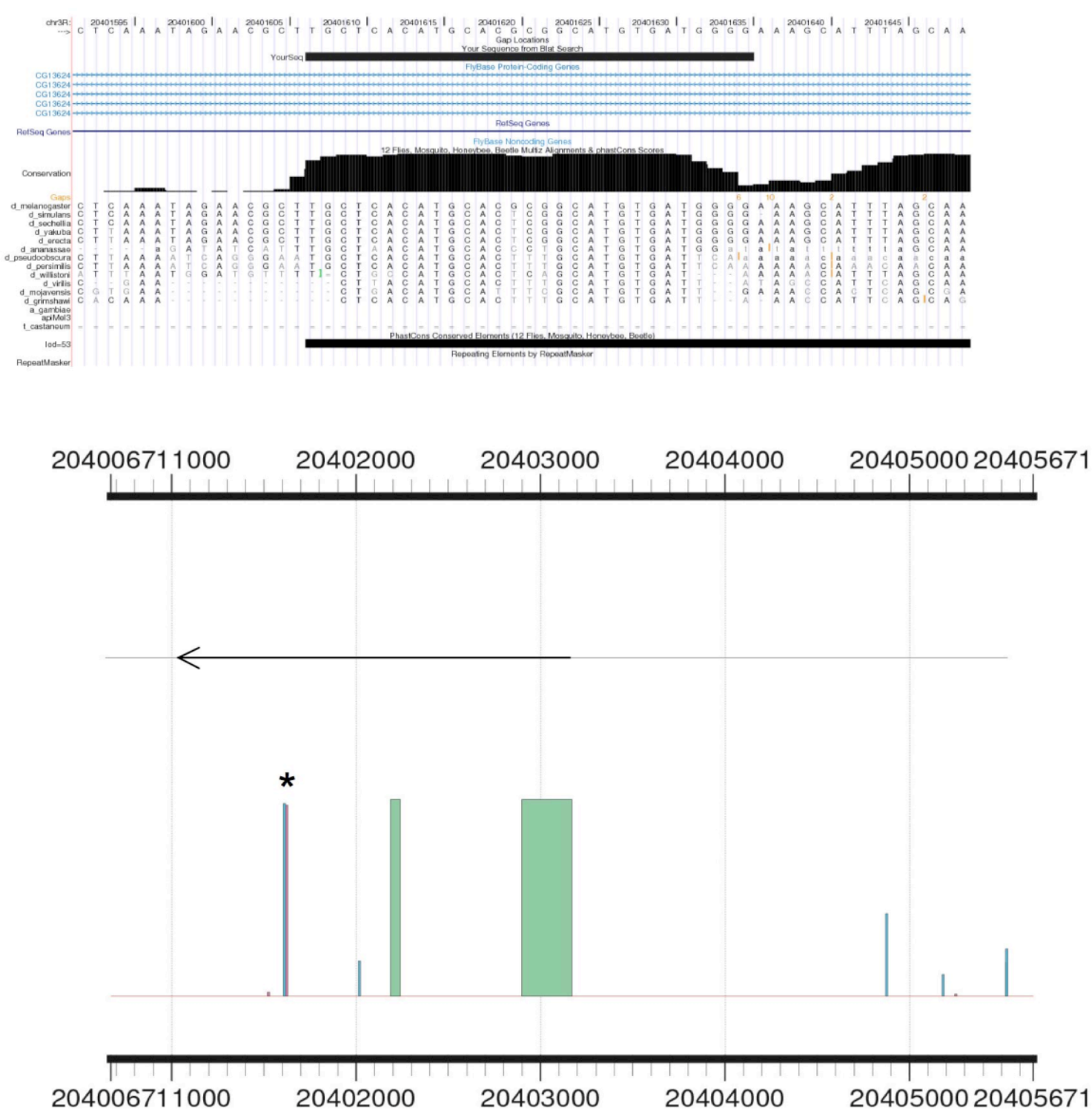

Fig. S3G l(2)08717

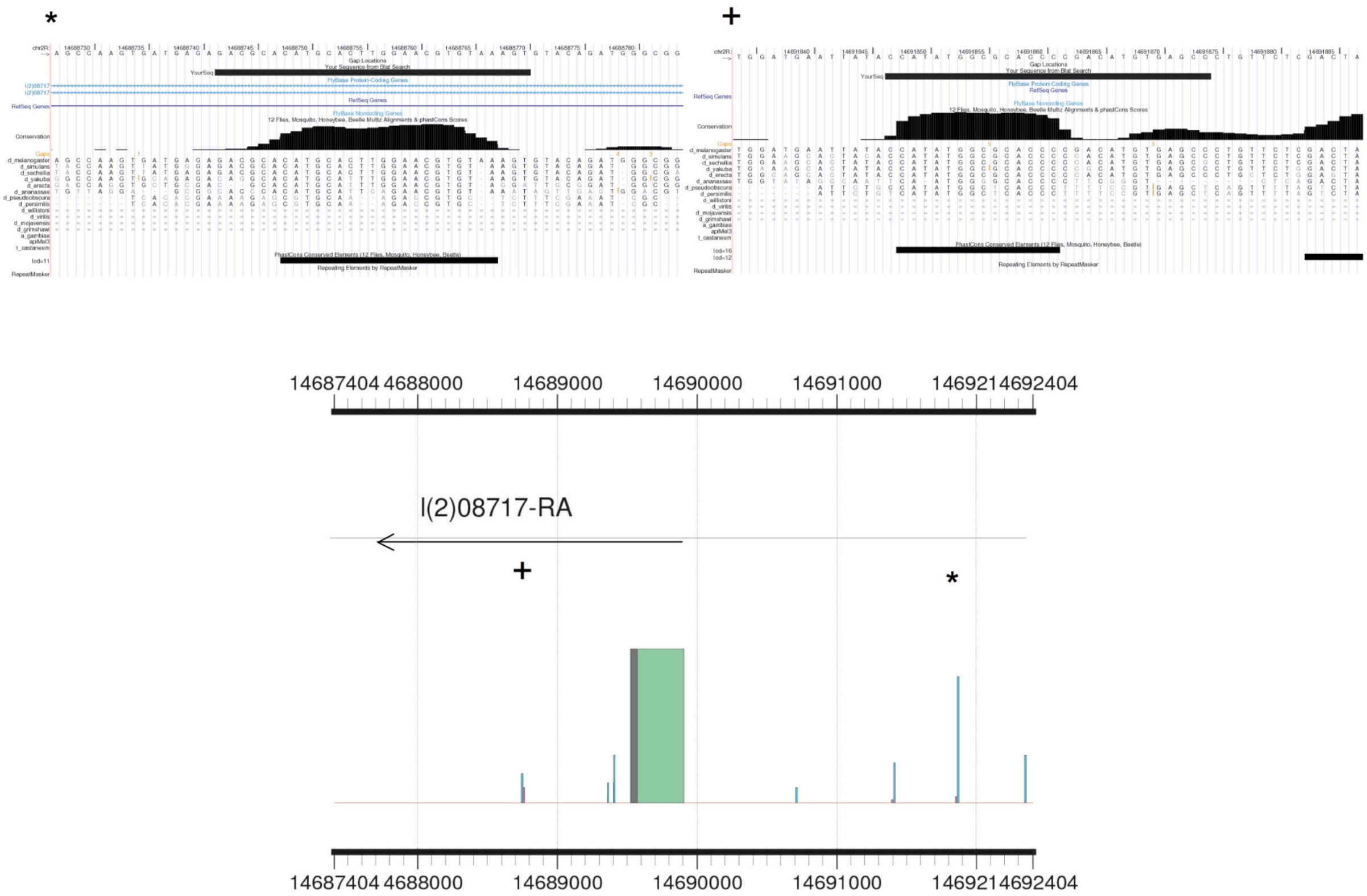

Figure S3. Positions of E1 and E2 sites around TSSs of circadian genes in flies.

Positions of E1 and E2 hits around TSSs. Major ticks always represent 1kb, 5 'UTR sequence is shown as green boxes and coding exons as gray boxes. Transcripts are show in their native orientation, as indicated by the arrows. The E1 boxes are indicated by cyan bars and the E2 boxes by magenta bars. The heights of the bars represent the posterior probabilities of the E1 and E2 boxes (Methods). The maximum of 1 is given by the size of the exons (we modified the gff2ps_v0.98d program to have bar heights which are truly proportional to the posterior probabilities). '*, +', or '&' on the upper panels indicate the positions of the zoomed panels (bottom). **A-E**: Instances of E1 and E2 boxes in the 5 training genes. **F-G**: Instances of E1 and E2 boxes for 2 genes (*CG13624* and *l(2)08717*) showing strong induction in the GR-CLK experiment. Conservation profiles suggest that the combined E1-E2 locus is under negative selection constraint.

References :

1. Hao H, Allen DL, Hardin PE (1997) A circadian enhancer mediates PER-dependent mRNA cycling in Drosophila melanogaster. Mol Cell Biol 17: 3687-3693.
2. McDonald MJ, Rosbash M, Emery P (2001) Wild-type circadian rhythmicity is dependent on closely spaced E boxes in the Drosophila timeless promoter. Mol Cell Biol 21: 1207-1217.
