# Supplementary material for: Modeling an Evolutionary Conserved Circadian Cis-Element
Source: PLoS Comput Biol. 2008 Feb 15;4(2):e38. doi: 10.1371/journal.pcbi.0040038 (PMC2242825; doi:10.1371/journal.pcbi.0040038)
Supplement: Supplementary file 4 [file pcbi.0040038.sg004.pdf]

Supplementary material to E. Paquet, G. Rey, and F. Naef (PLoS Computational Biology).

Fig. S4 A

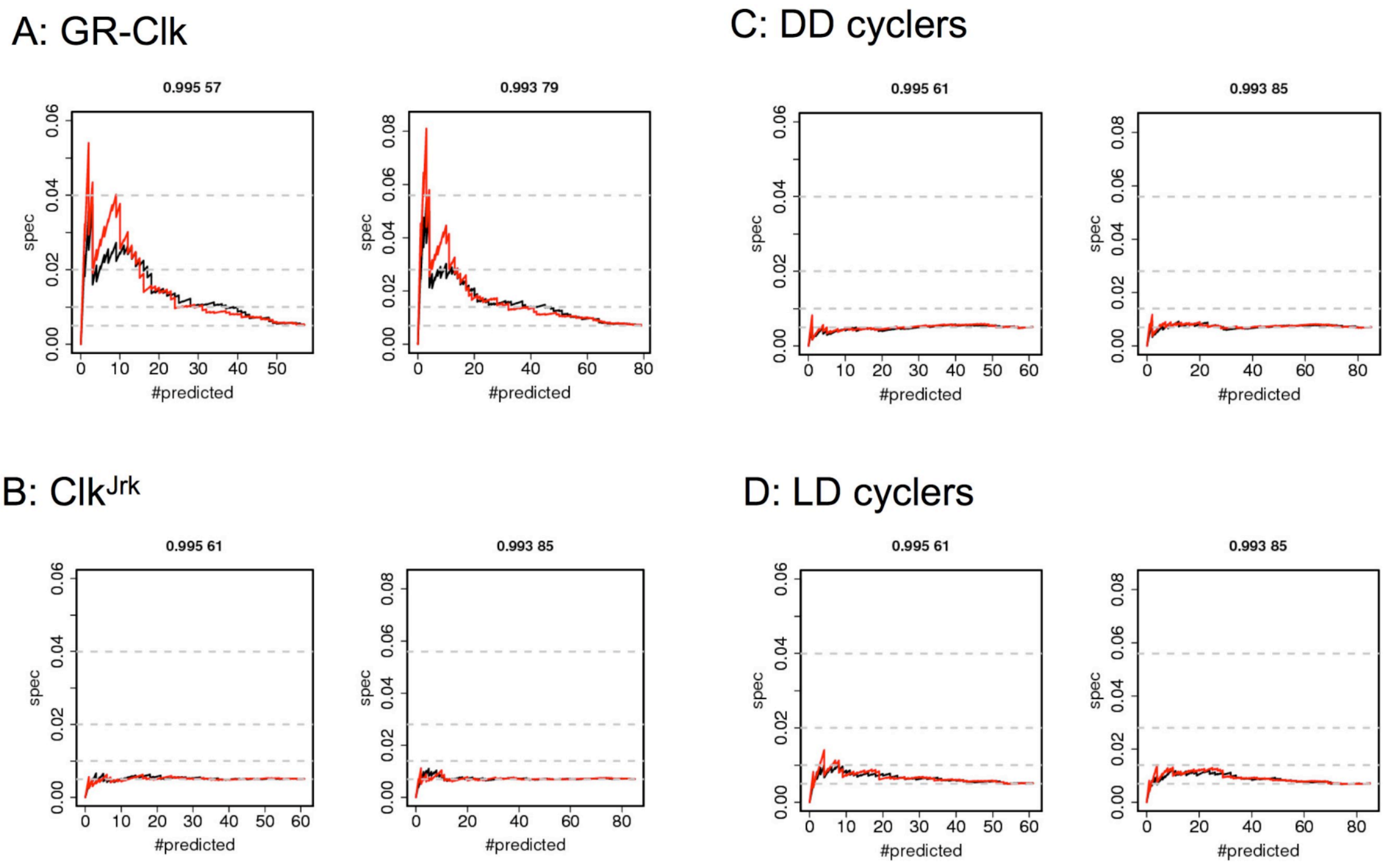

Fig. S4 B

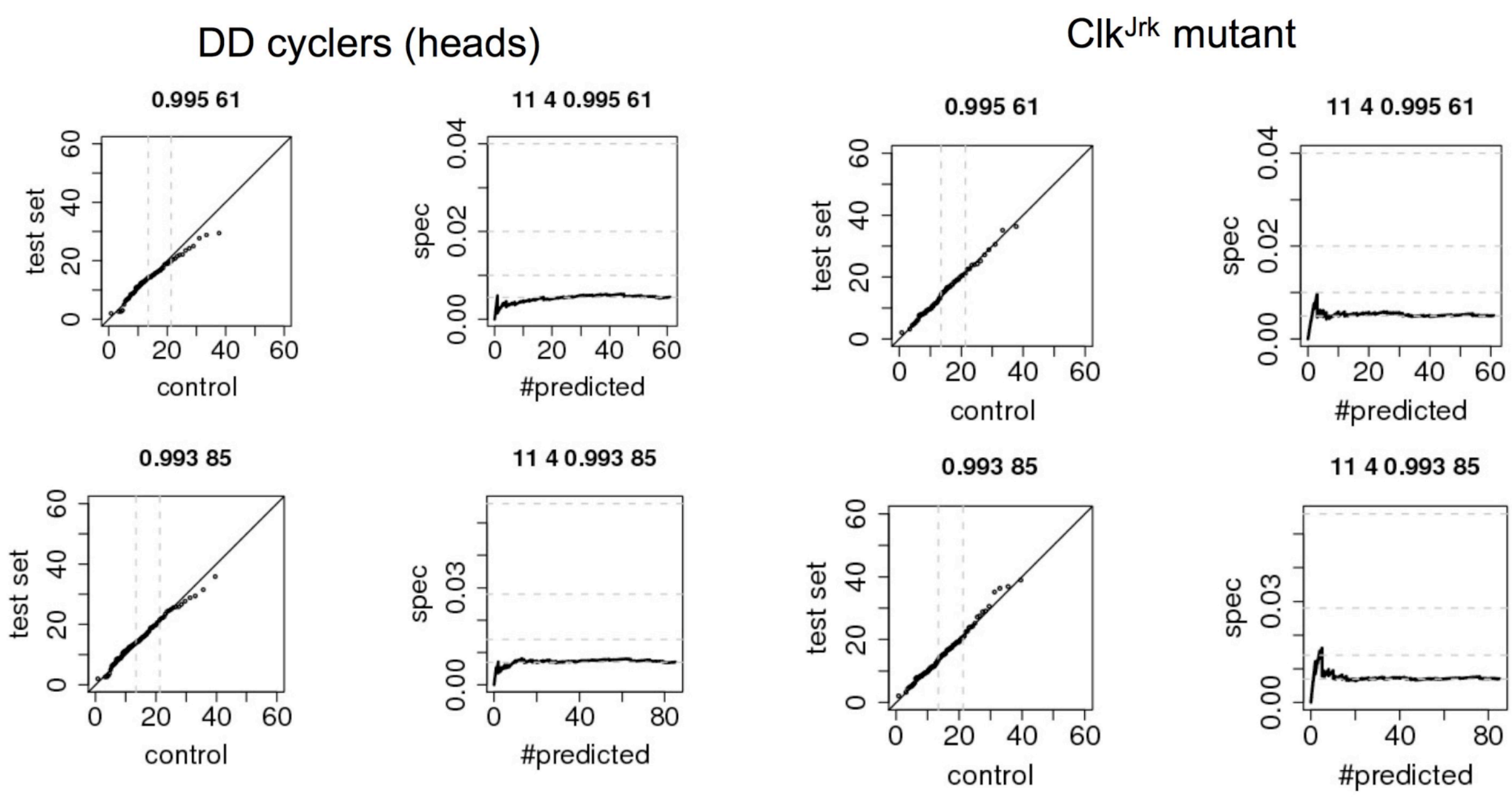

Figure S4. In Drosophila E1-E2 signal is not enriched in cyclic genes or genes which are downregulated in *Clock*^{Jrk} flies.

A: Specificities of the E1-E2 signal in four different experiments and comparison of E1-E2 model (black) with E1 model alone (red). The top 0.5% (and 0.7% genes) in the GR-CLK induction experiment, down-regulation in *Clock*^{Jrk} mutants, and cycling (measured as 24h Fourier scores) in

entrained LD (light-dark) or free-run DD (dark-dark) conditions, are used to define the set of positives (Methods). Only the GR-CLK induction shows a sign of enriched sequence motifs. In this experiments the E2 box seems not to increase specificity much. A closer inspection shows that specificity in the presence of E2 is in fact reduced for low sensitivities (<10 predicted genes), but that the black and red curves crossover and that the black is slightly more specific for larger sensitivities. The five training genes are removed from this analysis.
B: Quantile-quantile plots of the scores and specificity assessment in $\textit{Clock}^{Jrk}$ repressed genes and DD cyclers for the E1-E2 model (compare with Fig. 3).
