# Supplementary material for: Modeling an Evolutionary Conserved Circadian Cis-Element
Source: PLoS Comput Biol. 2008 Feb 15;4(2):e38. doi: 10.1371/journal.pcbi.0040038 (PMC2242825; doi:10.1371/journal.pcbi.0040038)
Supplement: Supplementary file 5 [file pcbi.0040038.sg005.pdf]

Supplementary material to E. Paquet, G. Rey, and F. Naef (PLoS Computational Biology).

Fig. S5

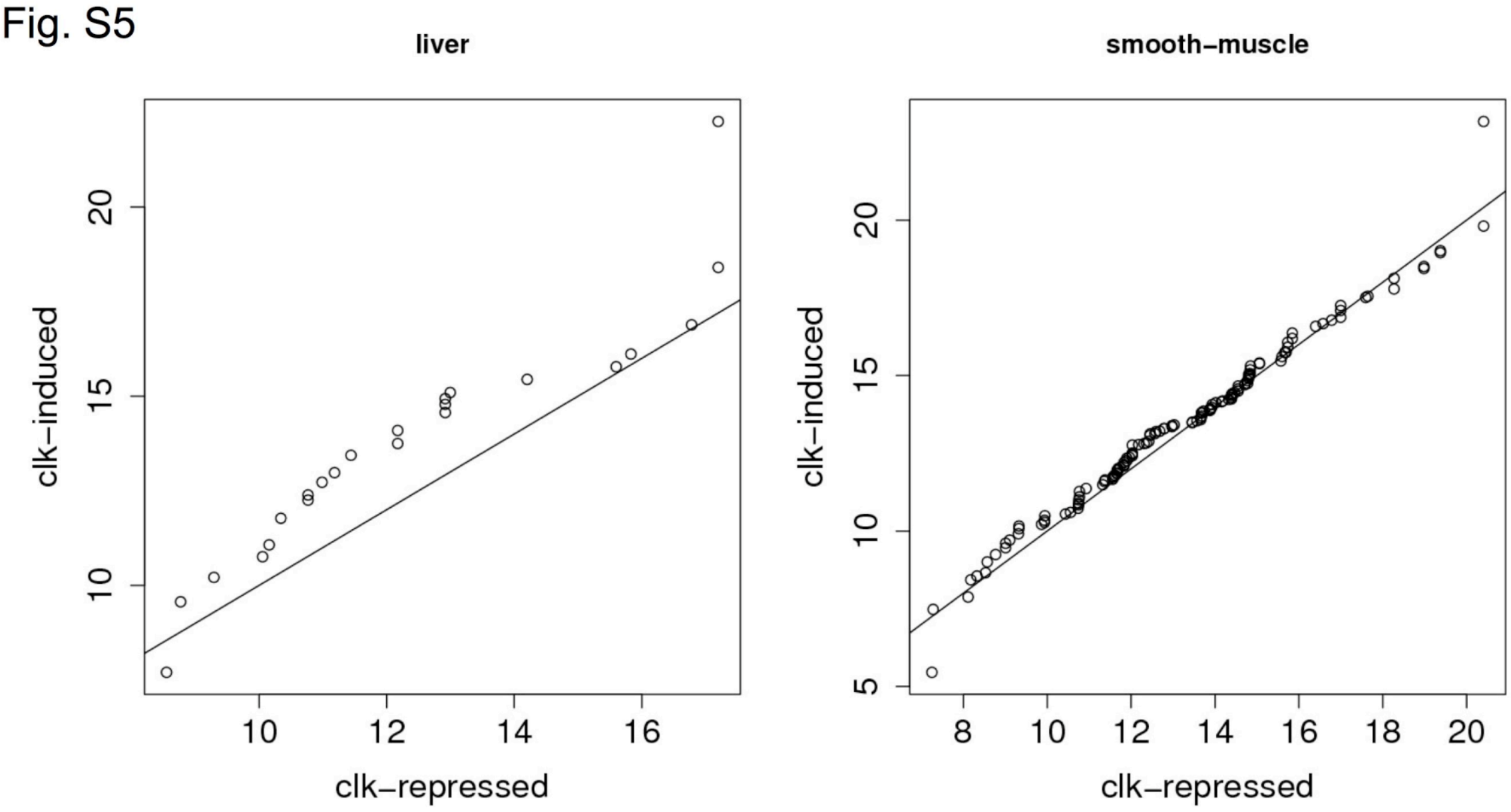

Figure S5. E1-E2 enhancers in Δ19 *Clock* mutants.

Specificity of the E1-E2 signal in *Clock* mutants in liver (left) and smooth muscle (right). CLOCK/BMAL1-induced or repressed genes are taken form the supplementary material in (Miller, 2007). While the liver samples show a weak tendency for CLOCK/BMAL1-induced genes to show stronger E1-E2 sites, the smooth muscle data does not discriminate between repressed or induced genes.

<u>References</u>

Miller BH, McDearmon EL, Panda S, Hayes KR, Zhang J, et al. (2007) Circadian and CLOCK-controlled regulation of the mouse transcriptome and cell proliferation. Proc Natl Acad Sci U S A 104: 3342-3347
