# Supplementary material for: Modeling an Evolutionary Conserved Circadian Cis-Element
Source: PLoS Comput Biol. 2008 Feb 15;4(2):e38. doi: 10.1371/journal.pcbi.0040038 (PMC2242825; doi:10.1371/journal.pcbi.0040038)
Supplement: Supplementary file 6 [file pcbi.0040038.sg006.pdf]

Supplementary material to E. Paquet, G. Rey, and F. Naef (PLoS Computational Biology).

Fig. S6

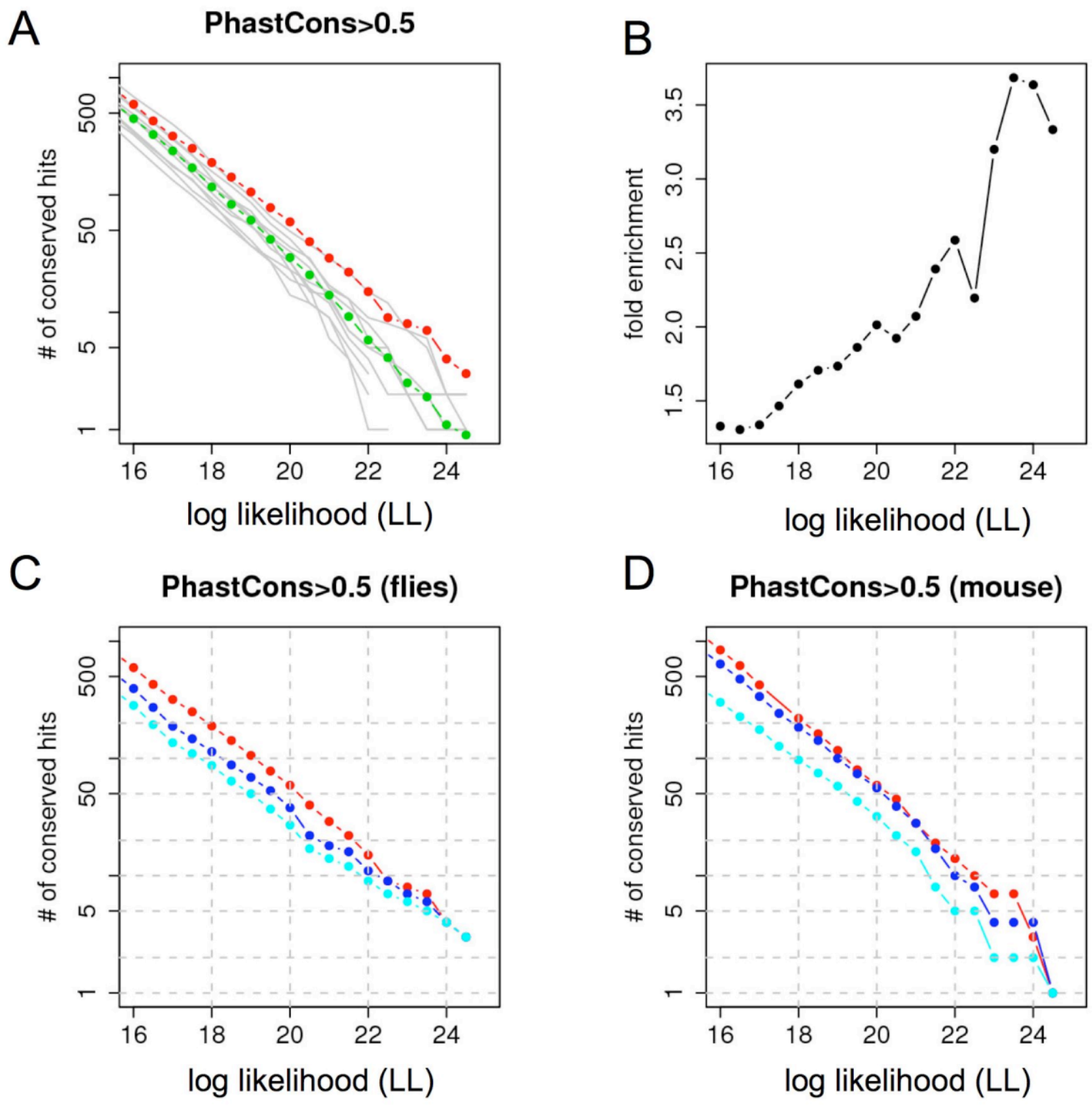

Figure S6. Estimated number of E1-E2 sites in flies and mouse.

A. Number of conserved hits on the D. Melanogaster genome with PhastCons score higher than 0.5 in function of the likelihood score (x-axis). We show the original E1-E2 matrices (red) and ten randomly permutated versions (gray lines) with their average (green).

B. Enrichement observed for the real E1-E2 matrix as compared to the mean of the permuted models from A (red divided by green).

C. *D. melanogaster*: Number of conserved (PhastCons>0.5, from genome version dm3, 15 species) hits anywhere (red), in the +/-2.5kb regions around TSSs (blue), and number of genes with at least one hit in the TSS region (cyan).

D. Mouse: number of conserved (PhastCons>0.5, mm8 17 species) hits anywhere (red), in the +/-2kb regions around genes (from the MOE430 table, cf. Methods) (blue), and in smaller regions consisting of +/- 2kb around the TSS (cyan).
