# Supplementary material for: Modeling an Evolutionary Conserved Circadian Cis-Element
Source: PLoS Comput Biol. 2008 Feb 15;4(2):e38. doi: 10.1371/journal.pcbi.0040038 (PMC2242825; doi:10.1371/journal.pcbi.0040038)
Supplement: Supplementary file 7 [file pcbi.0040038.sg007.pdf]

Supplementary material to E. Paquet, G. Rey, and F. Naef (PLoS Computational Biology).

Fig. S7A

Rev-erbα

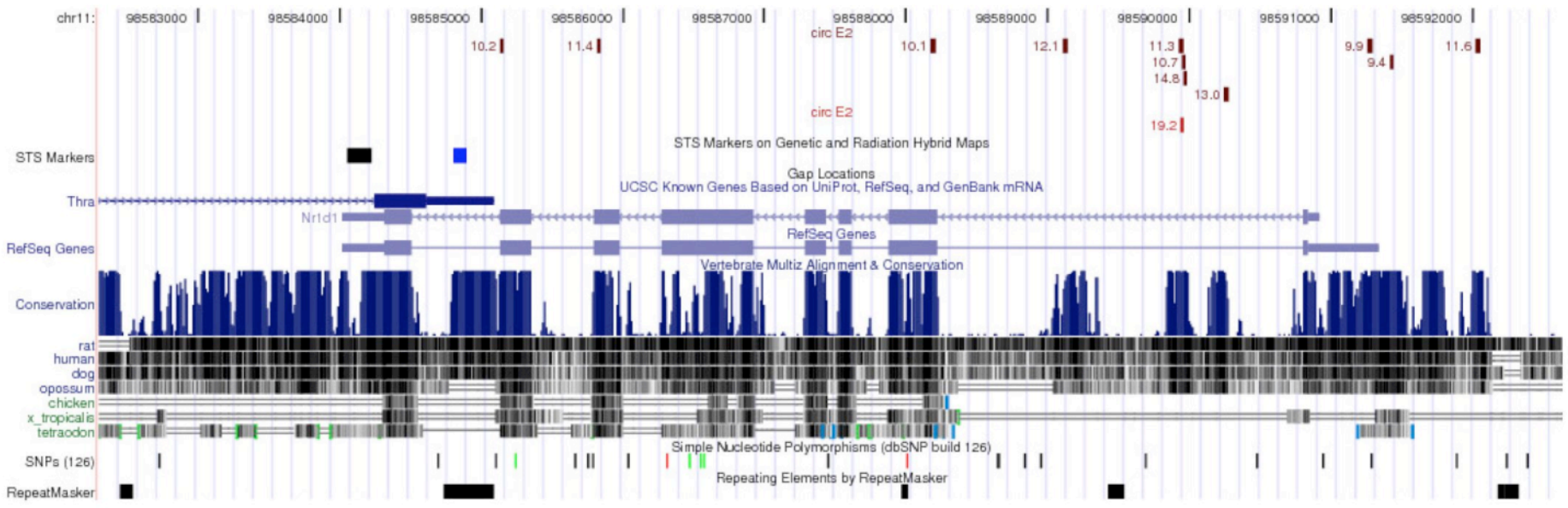

1rst intron

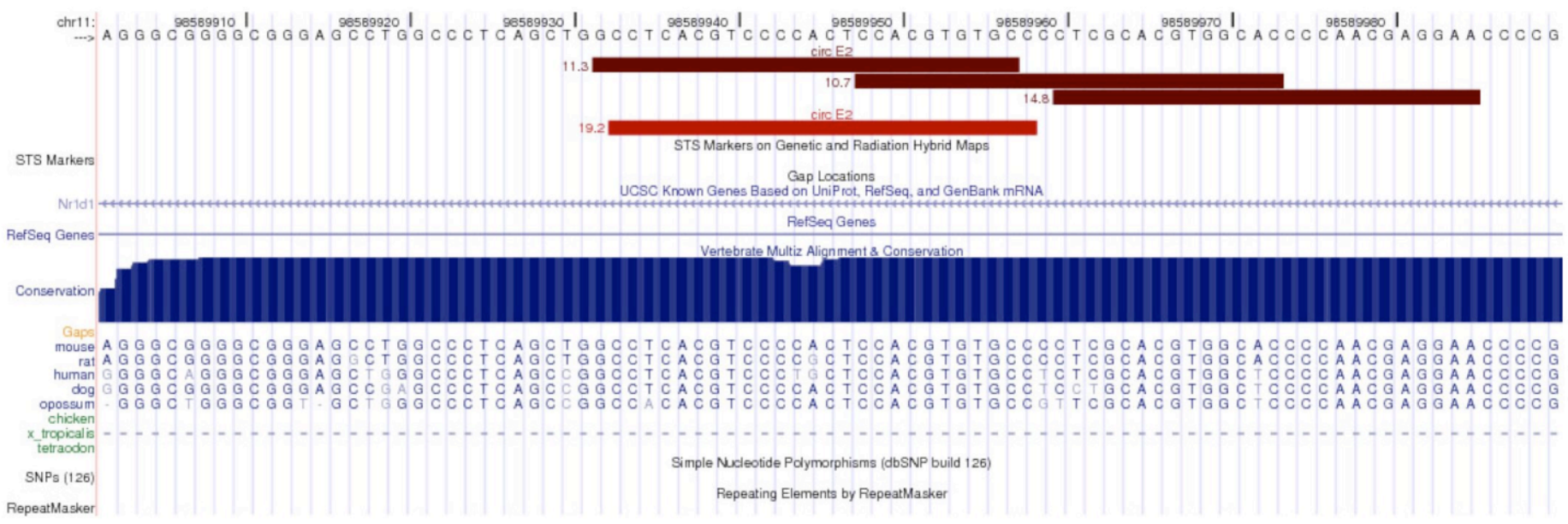

Fig. S7B

Per1

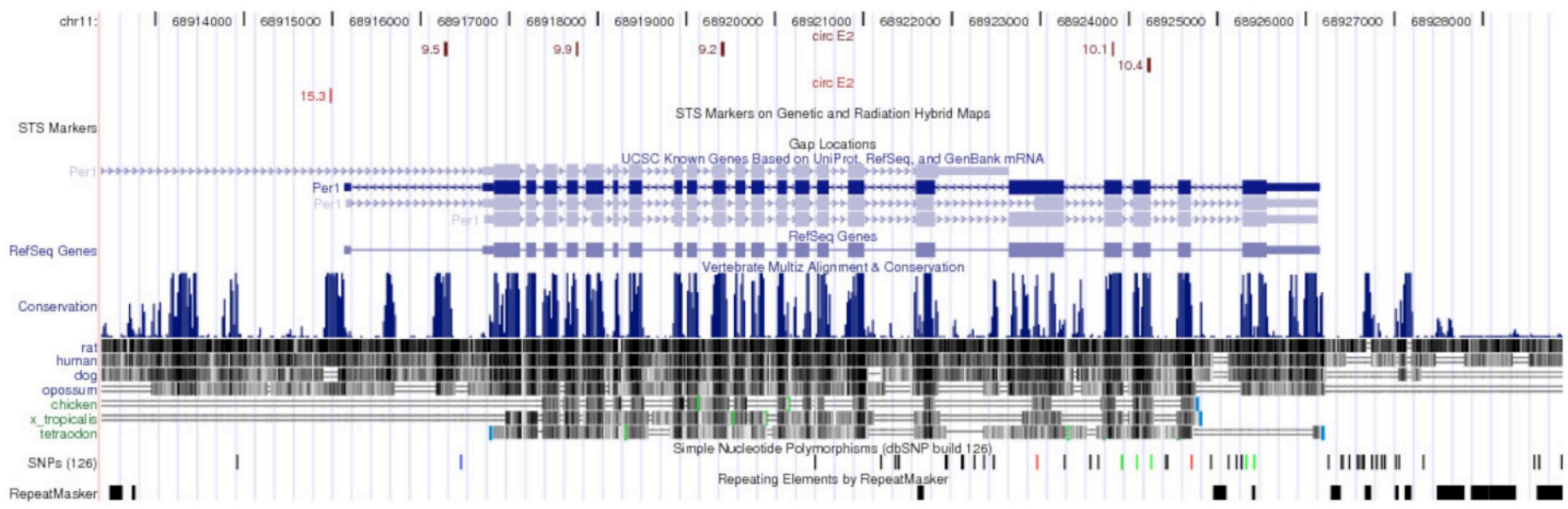

promoter

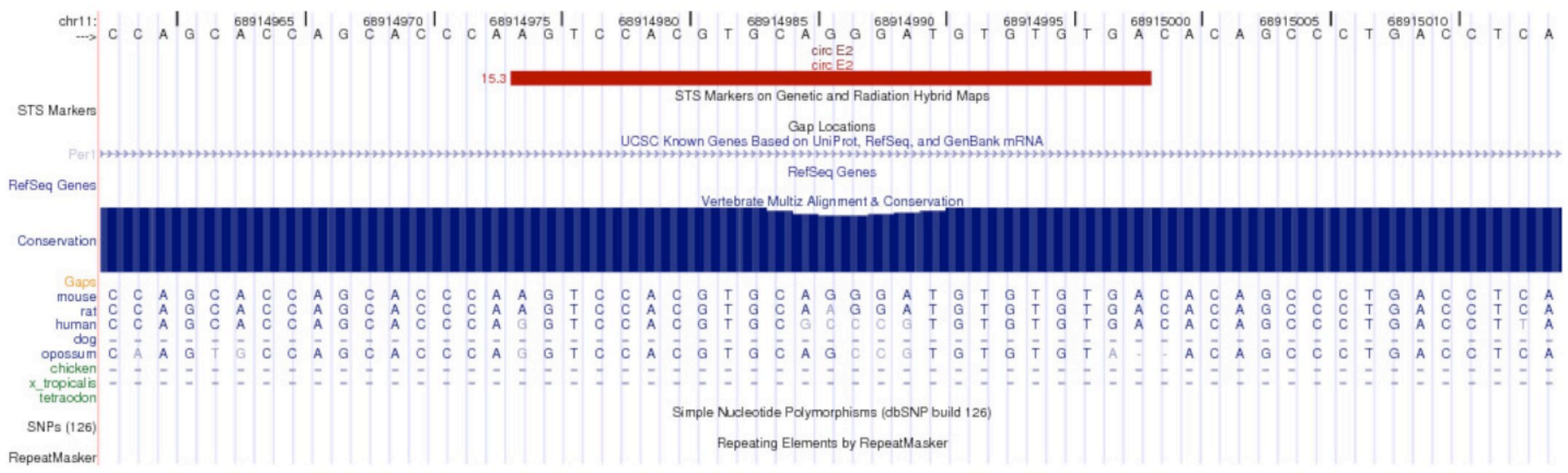

Fig. S7C

Per2

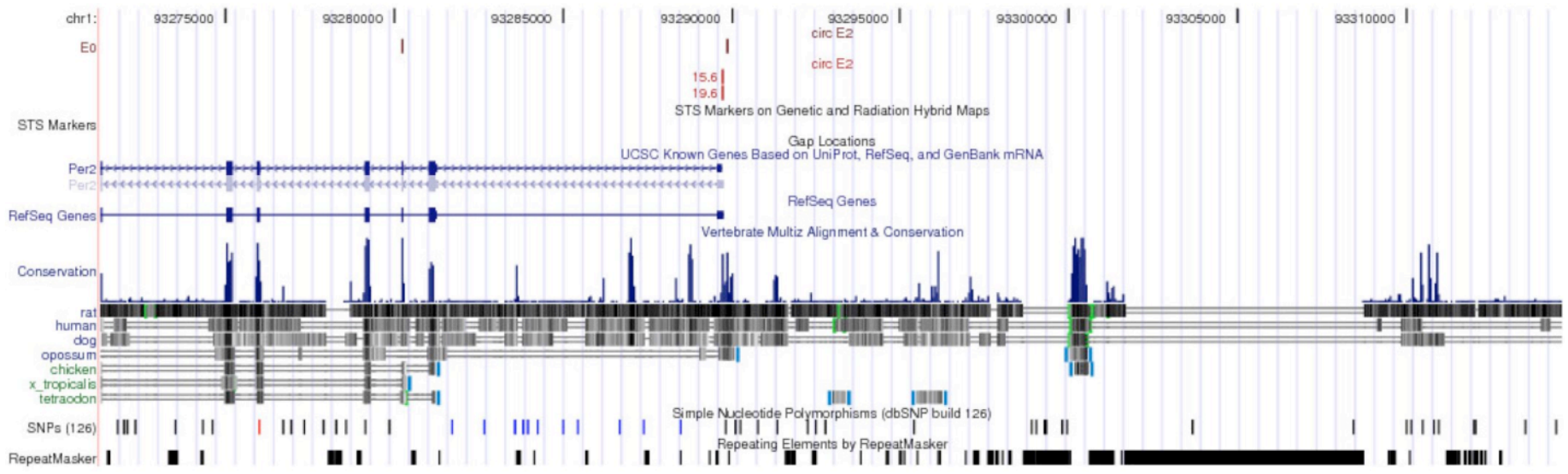

promoter

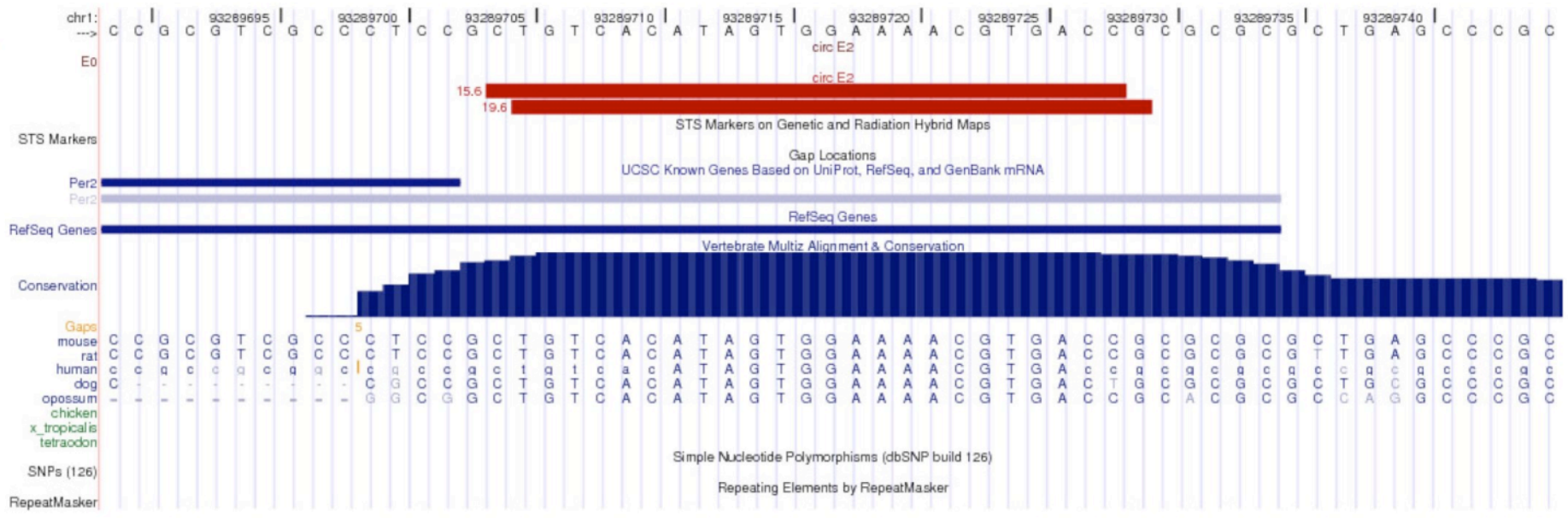

Fig. S7D

Cry2

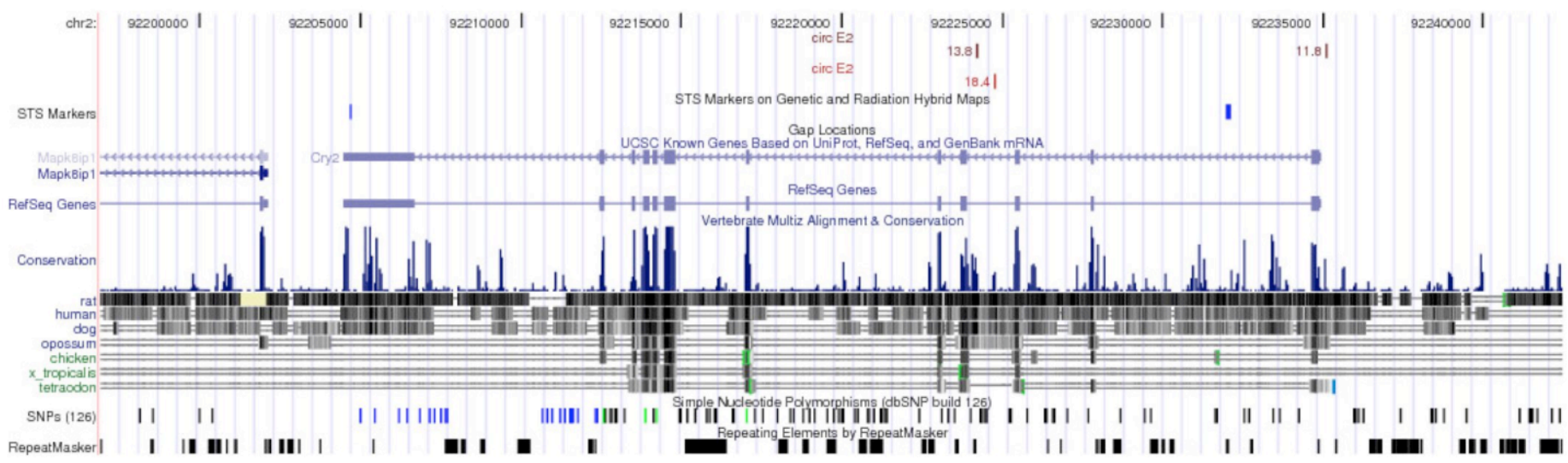

promoter

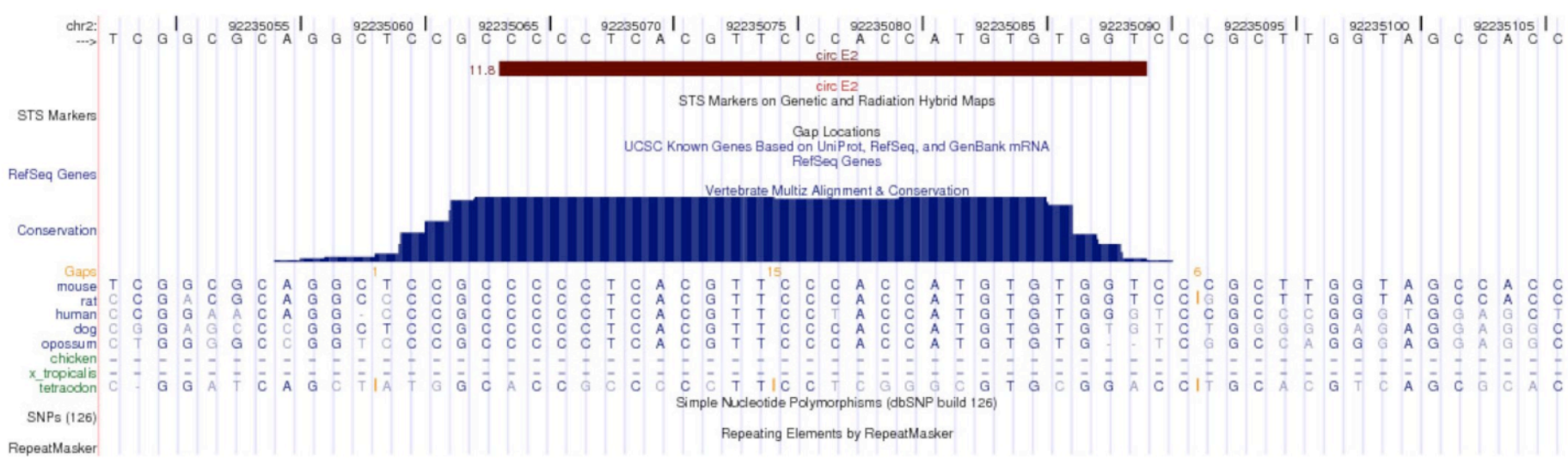

intron 3

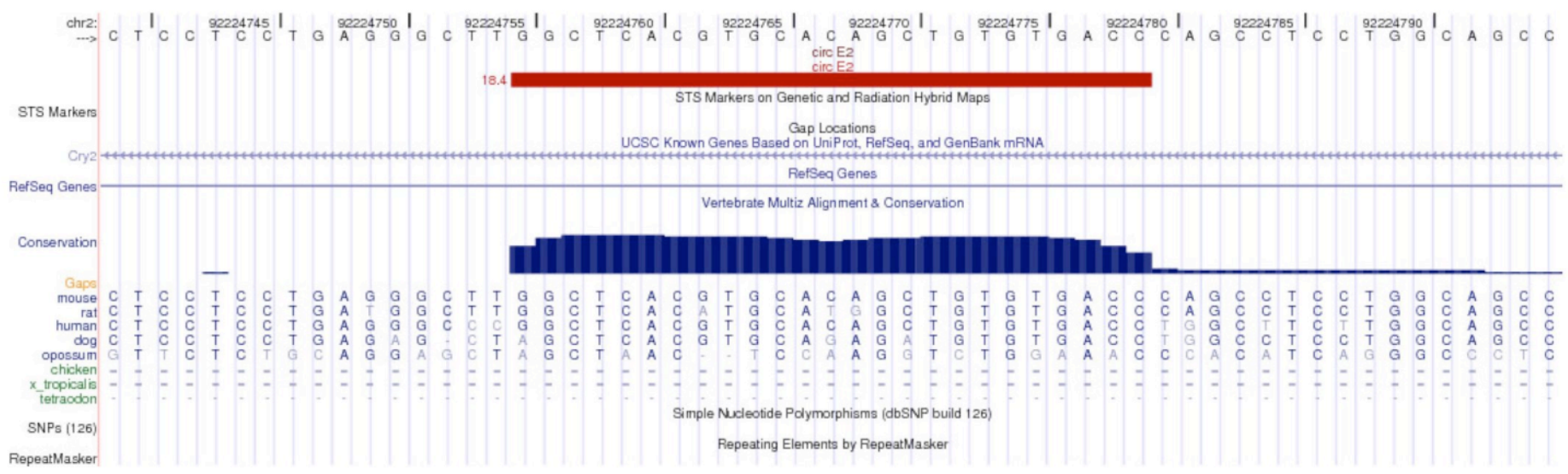

Fig. S7E

Dbp

promoter

intron 1

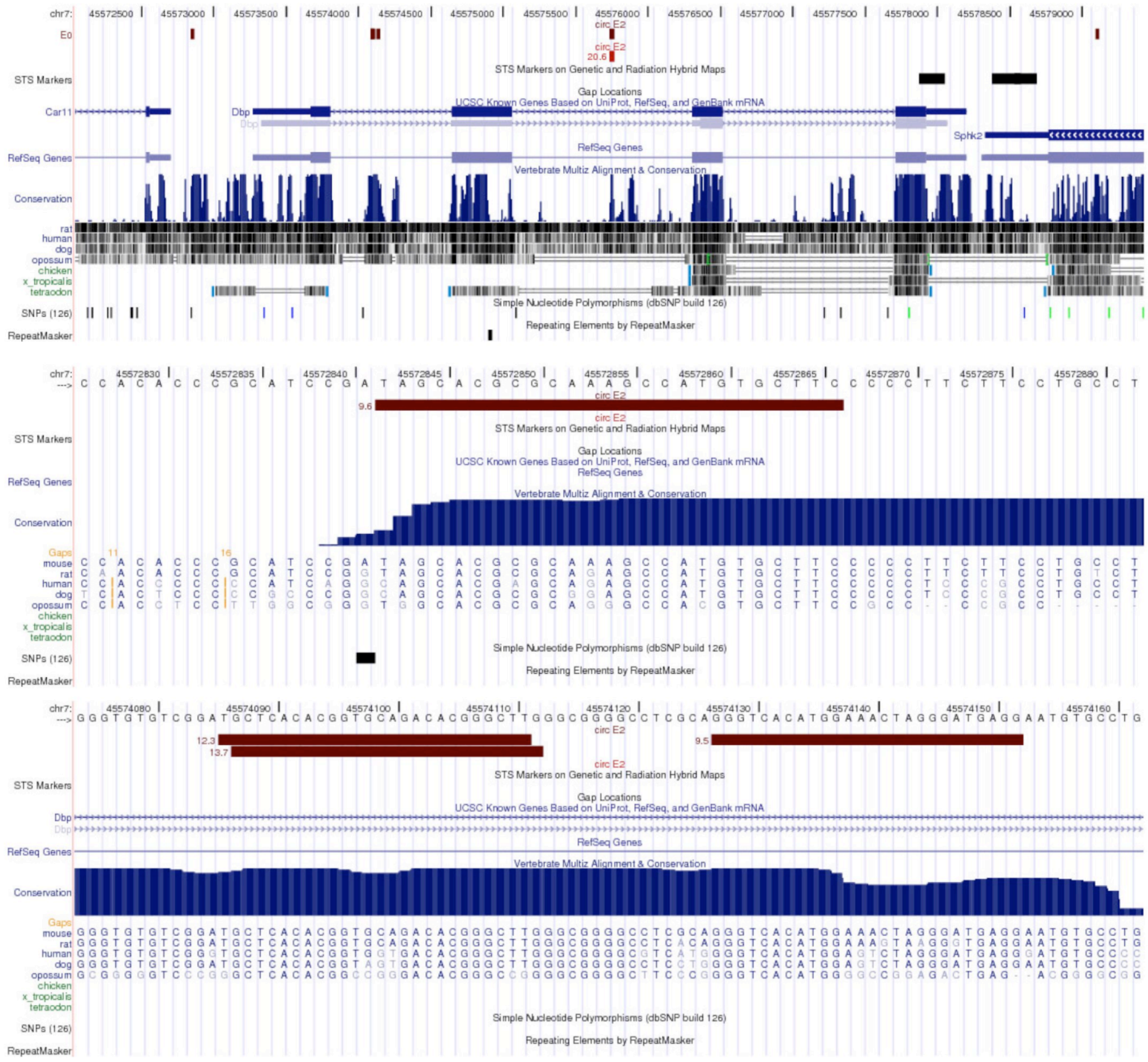

Dbp intron 2

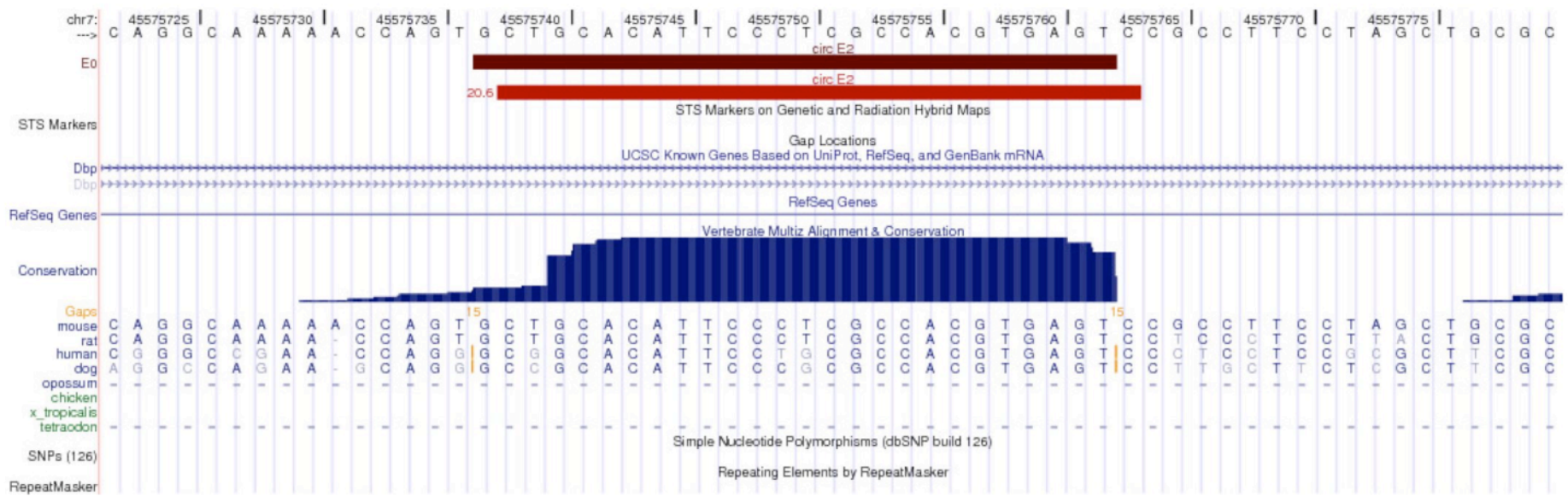

Fig. S7F

Wee1

3’ end

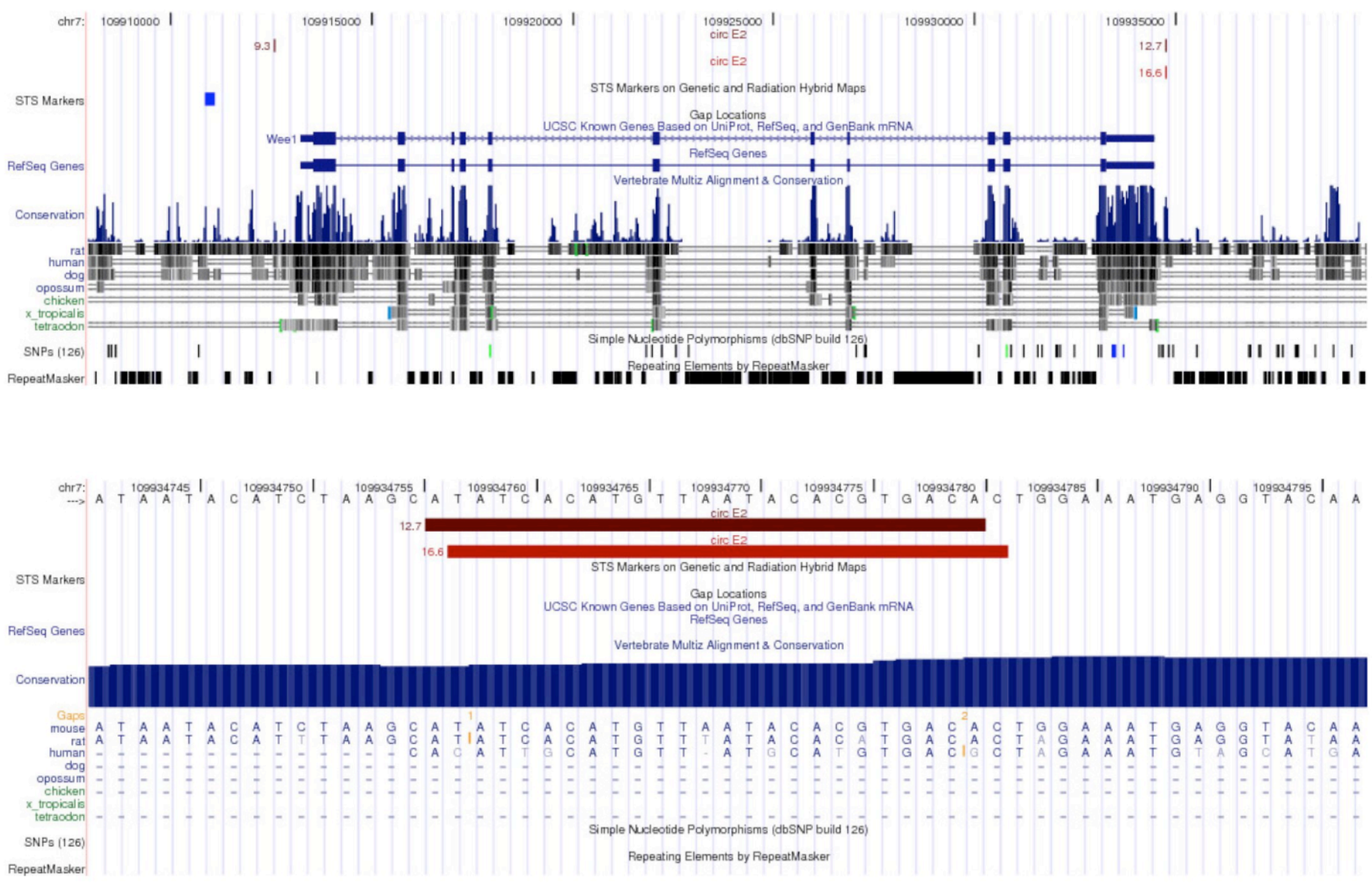

Fig. S7G

Tef

promoter

promoter

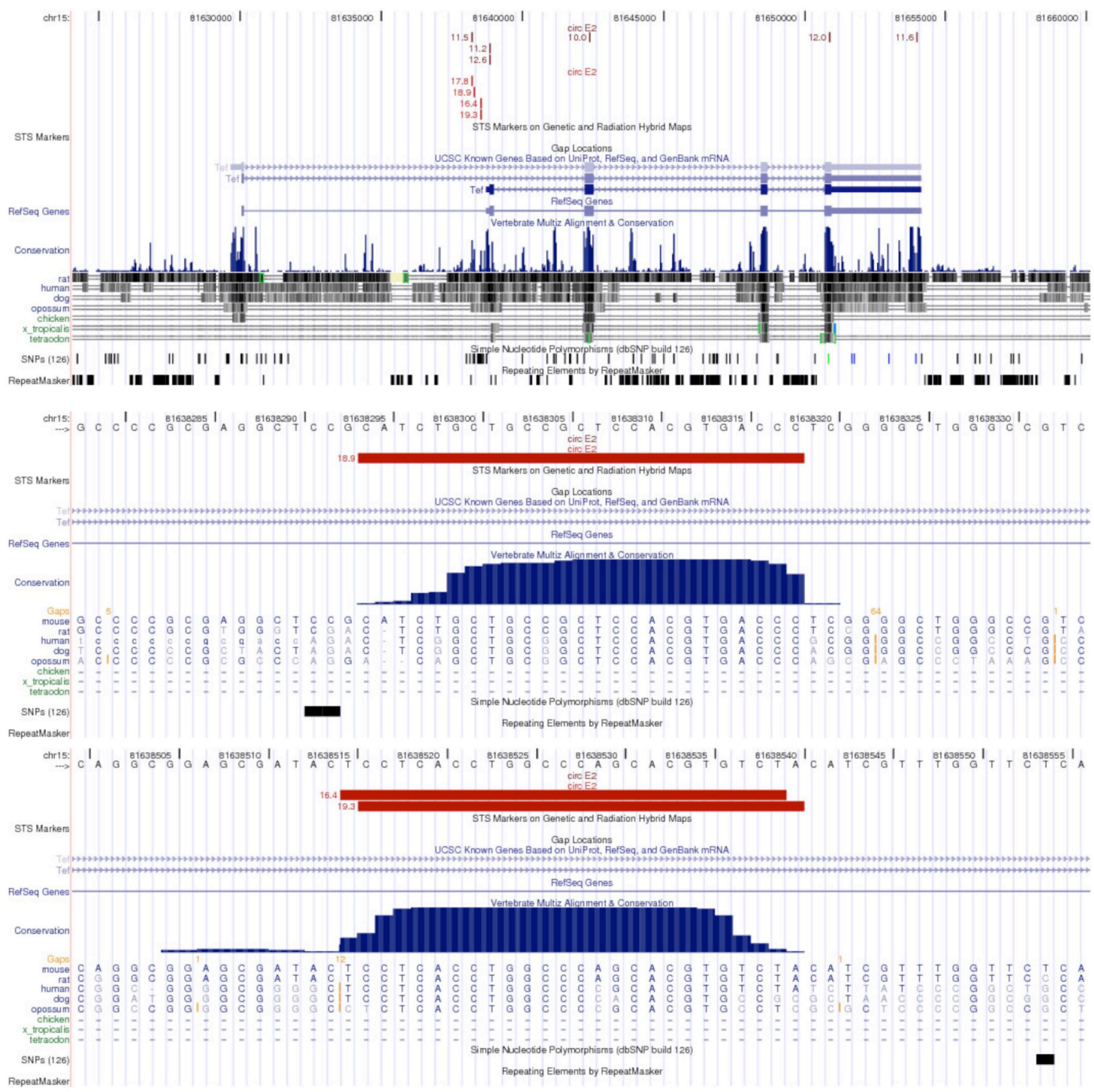

Fig. S7H

Hlf

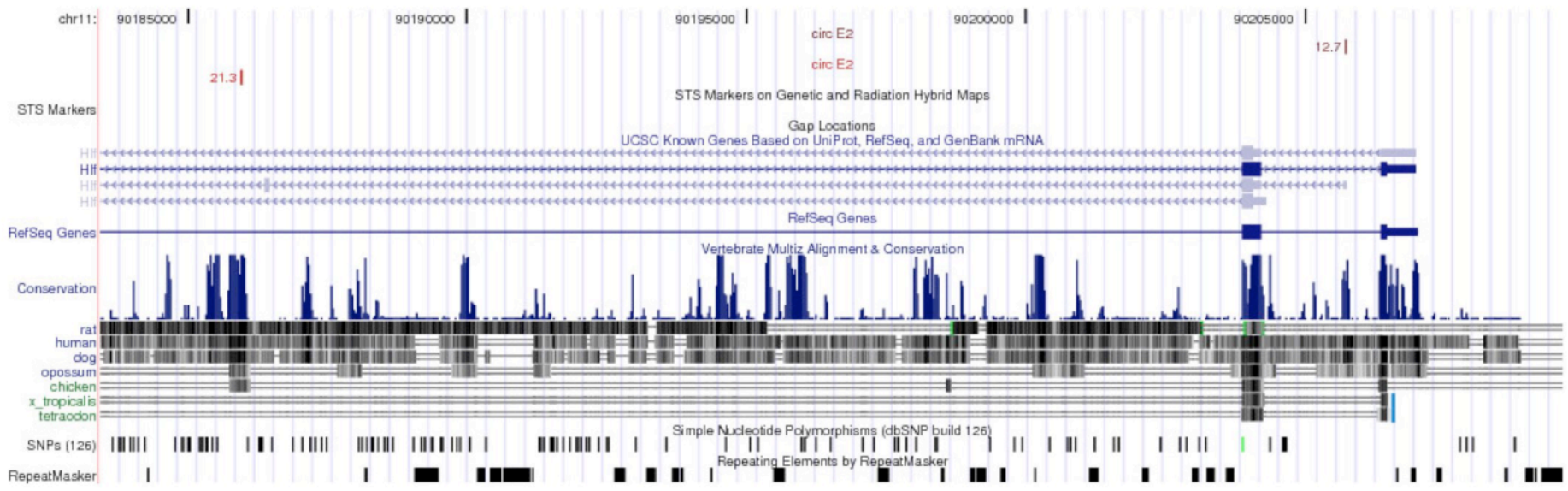

intron 1

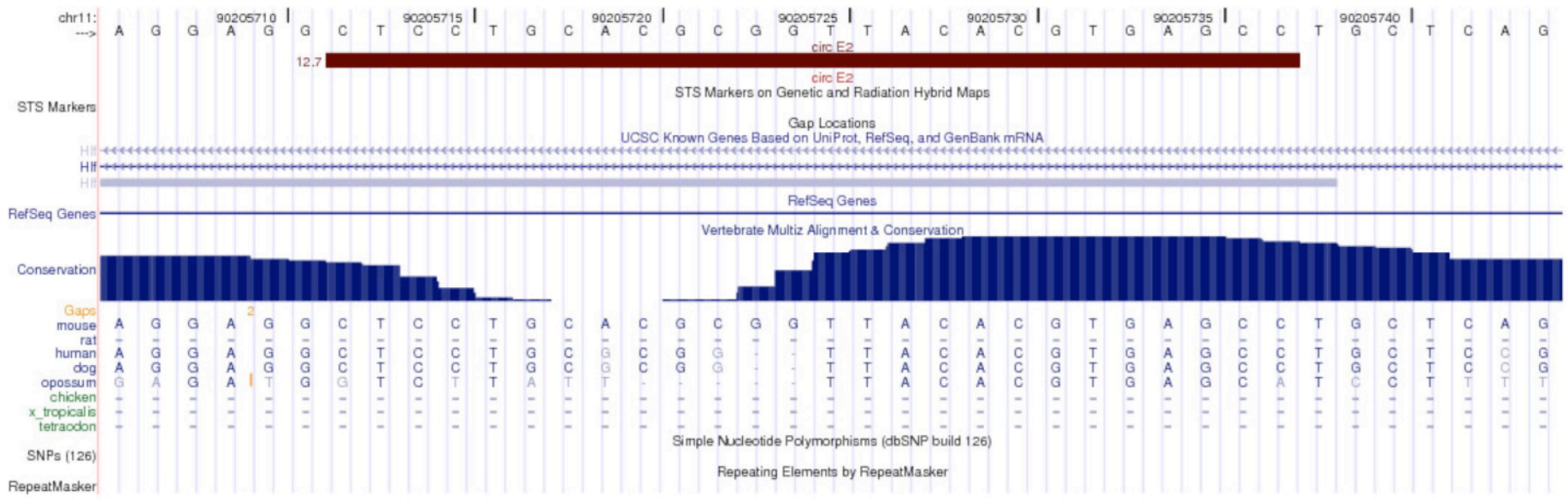

intron 2

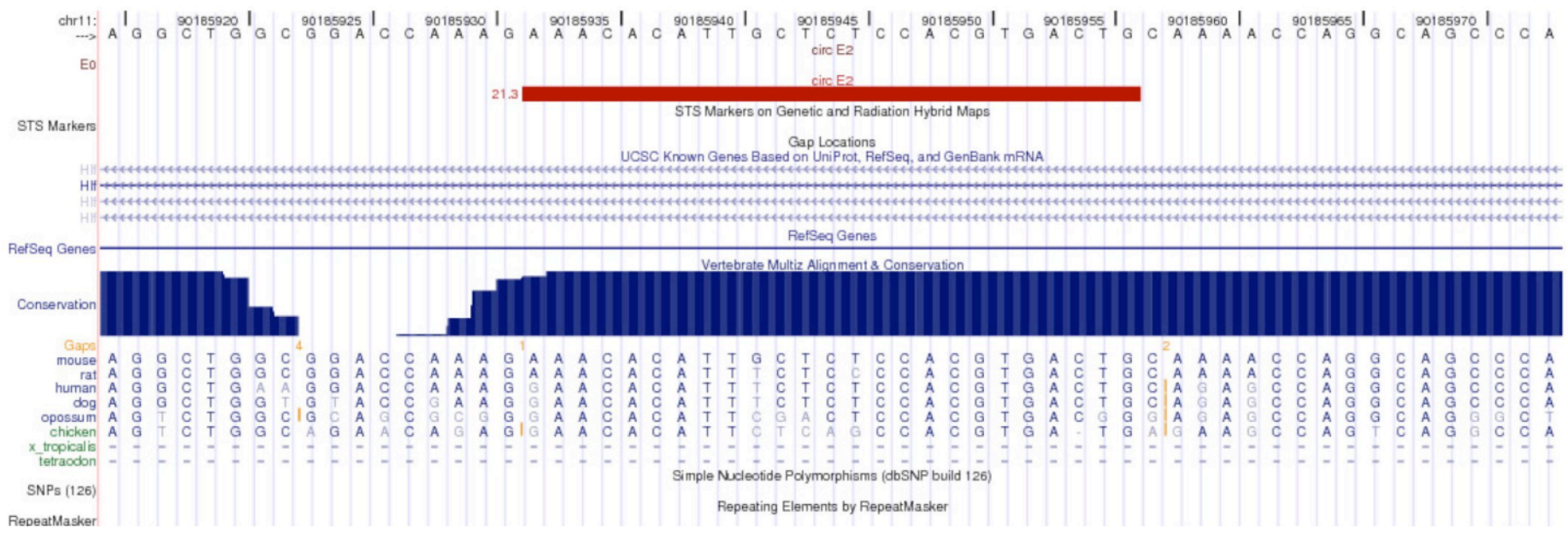

Fig. S7I

Bhlhb2

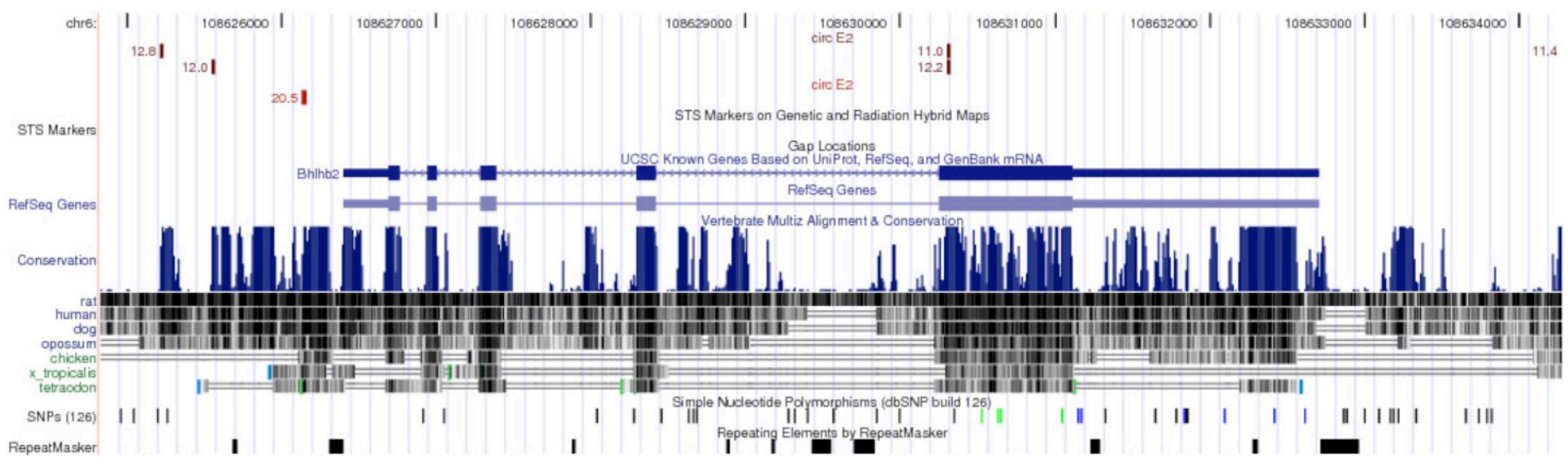

promoter

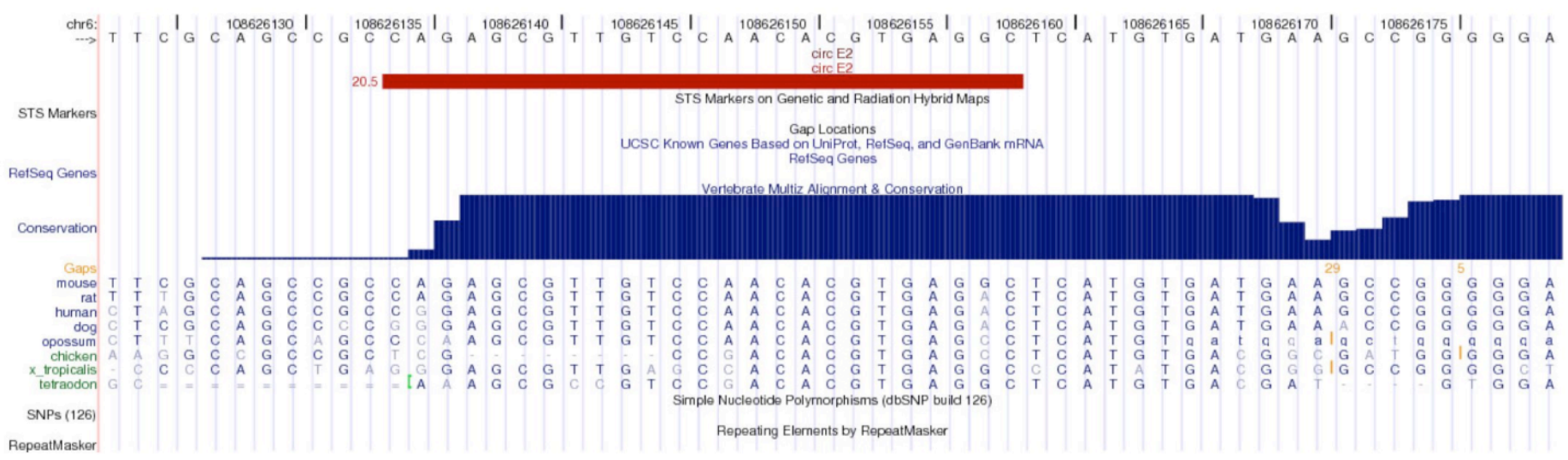

Figure S7. Position of E1-E2 enhancers in circadian mouse genes.

Plots are from the UCSC genome browser with our hit file (available as mouse-scan_conserved.bed) uploaded as a custom track. Hits with score between 9-15 bits are shown in dark red and hits above 15 bits in bright red. For each gene we show a large-scale overview and zoomed views that emphasize the conservation profiles. Since the E1-E2 sequence is nearly palindromic, we often find hits that are doubled but offset by one base (e.g. the Per2 gene, panel C). Alignments are from the UCSC mm8 genome version.
