# Supplementary material for: Modeling an Evolutionary Conserved Circadian Cis-Element
Source: PLoS Comput Biol. 2008 Feb 15;4(2):e38. doi: 10.1371/journal.pcbi.0040038 (PMC2242825; doi:10.1371/journal.pcbi.0040038)
Supplement: Supplementary file 8 [file pcbi.0040038.sg008.pdf]

Supplementary material to E. Paquet, G. Rey, and F. Naef (PLoS Computational Biology).

Fig. S8

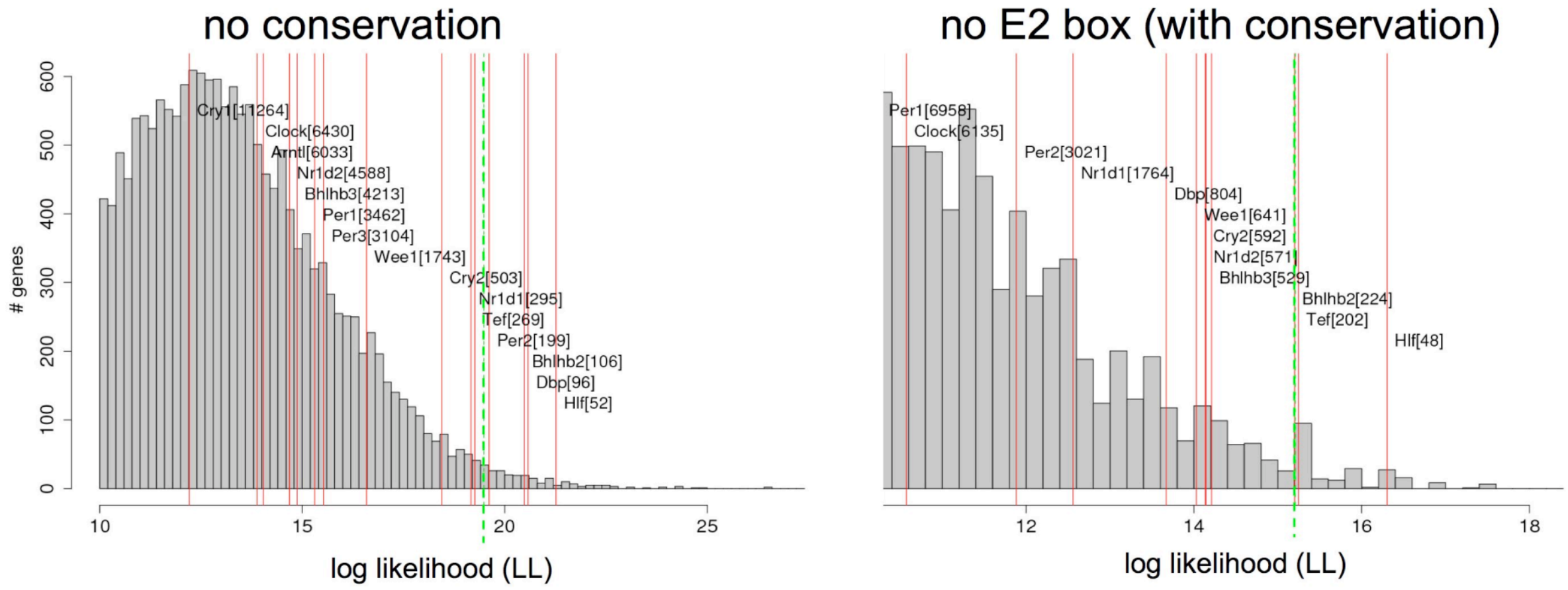

Figure S8. Sequence conservation and E2 improve the specificity of E1-E2 sites in mouse. Effect of sequence conservation (A) and E2-box (B) on the genome-wide rankings of circadian genes. 4 genes pass the 1% line (in green) when no sequence conservation is imposed (A). 3 genes pass the same line when only the E1 box is used in the model (B). The name of the gene is always aligned to the right of its score line (in red), and its rank is indicated in brackets.
