# Supplementary material for: Modeling an Evolutionary Conserved Circadian Cis-Element
Source: PLoS Comput Biol. 2008 Feb 15;4(2):e38. doi: 10.1371/journal.pcbi.0040038 (PMC2242825; doi:10.1371/journal.pcbi.0040038)
Supplement: Supplementary file 9 [file pcbi.0040038.sg009.pdf]

Supplementary material to E. Paquet, G. Rey, and F. Naef (PLoS Computational Biology).

Fig. S9

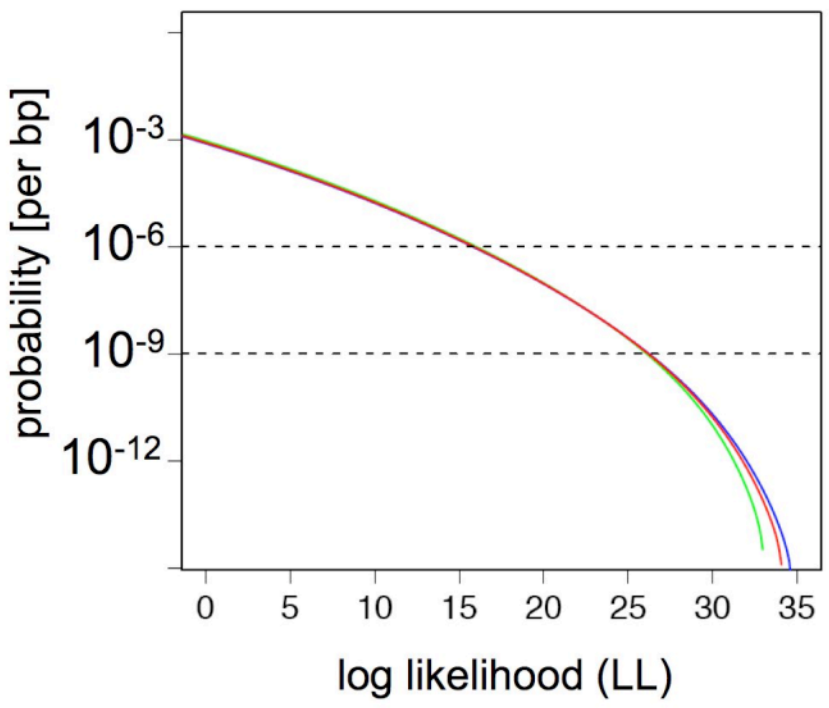

Figure S9. Background distribution for the likelihood (LL, cf. methods) score (in bits) of the E1-E2 model computed using the saddle point method as in [1]. The graph shows the per site probability to find a hit with score above the value in the x-axis. We considered single nucleotide background frequencies from the non-repetitive portions of the mouse (green), D. Melanogaster (blue) and fugu (red) genomes, which give very similar results. Agreement with empirical distributions is excellent.

Reference :

1. Djordjevic M, Sengupta AM, Shraiman BI (2003) A biophysical approach to transcription factor binding site discovery. Genome Res 13: 2381-2390.
