# Supplementary material for: Modeling an Evolutionary Conserved Circadian Cis-Element
Source: PLoS Comput Biol. 2008 Feb 15;4(2):e38. doi: 10.1371/journal.pcbi.0040038 (PMC2242825; doi:10.1371/journal.pcbi.0040038)
Supplement: Supplementary file 10 [file pcbi.0040038.sg010.pdf]

Supplementary material to E. Paquet, G. Rey, and F. Naef (PLoS Computational Biology).

Fig. S10

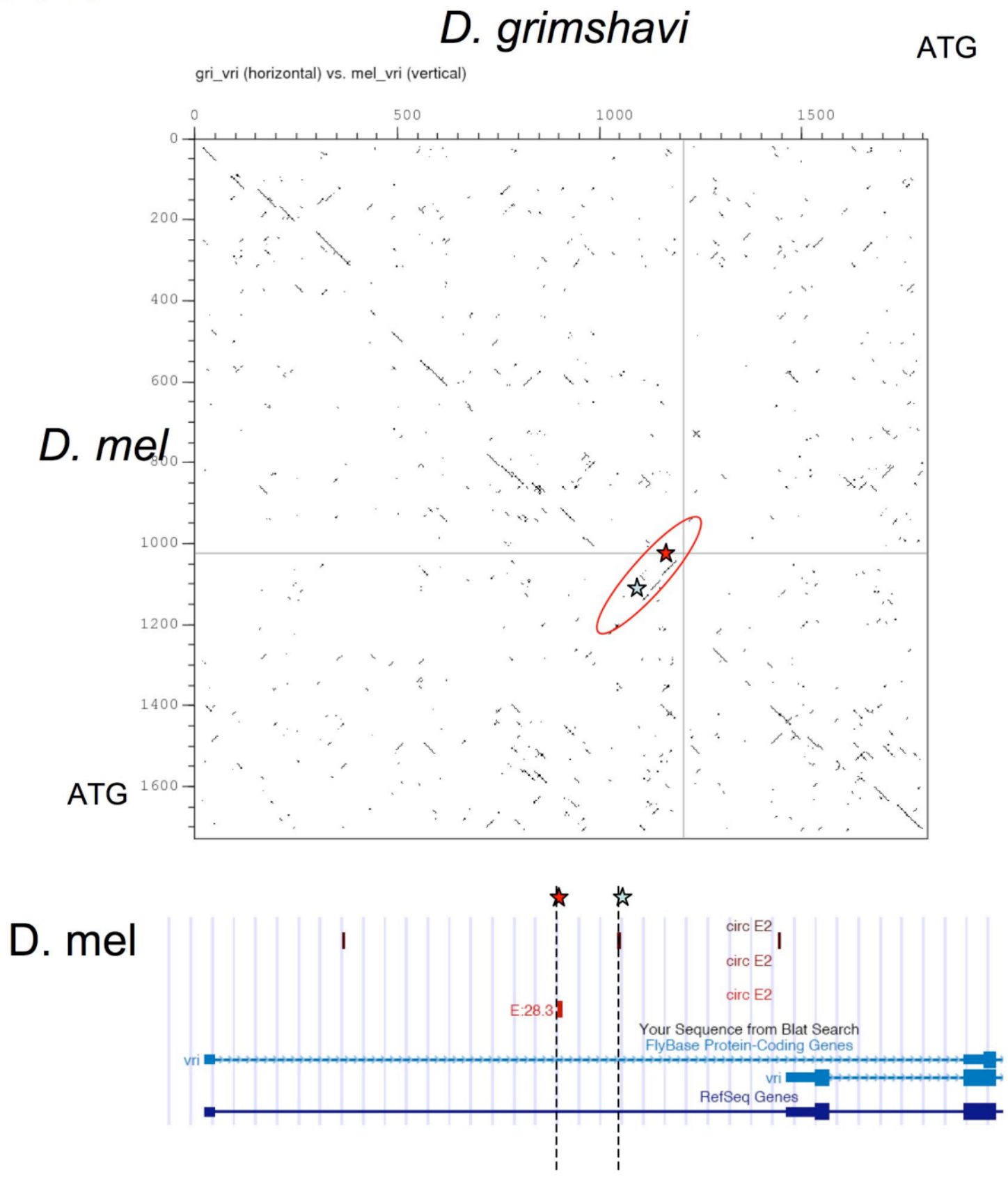

Figure S10. Sequence inversion in the *D. Grimshavi vrille* promoter.

A fragment in the *D. grimshawi vri* promoter harboring two conserved E1-E2 instances, (* and +, Fig. S2C) is inverted with respect to all other species. This explains why the Grimshawi sequence is missing form the UCSC multiple alignment in Fig. S2C.
