# Supplementary material for: Modeling an Evolutionary Conserved Circadian Cis-Element
Source: PLoS Comput Biol. 2008 Feb 15;4(2):e38. doi: 10.1371/journal.pcbi.0040038 (PMC2242825; doi:10.1371/journal.pcbi.0040038)
Supplement: Supplementary file 11 [file pcbi.0040038.st001.pdf]

Supplementary material to E. Paquet, G. Rey, and F. Naef (PLoS Computational Biology).

Table S1: Top 57 GR-CLK induced genes with annotations from sequence, 24 hours cycling in LD or DD, and regulation in *Clk*^{Jrk} data. The five training genes (green) are followed by top 57 induced genes in the GR-CLK experiment. The list of 57 is ordered according to the likelihood score of the E1-E2 model (2nd column). Appended are the LD and DD cycling scores F24 and phases (cf. Methods). F24 is maximally 1 for perfectly sinusoidal patterns. The last column contains the compiled fold change difference between *Clk*^{Jrk} and wild-type flies (Methods). Fields are highlighted when F24>0.15 (corresponds to p<0.03 in DD and p<0.04 in LD), or if the Clk fold induction is below -0.5, which corresponds to down regulation in the *Clk*^{Jrk} mutant. The list of top genes showing enrichment in Fig. 3 is marked in yellow.

	DrosGenome1	likelihood	Clk-GR fold induction	LD F24	LD phase	DD F24	DD phase	ClkJrk fold induction
per	143300_at	59.124	1.364	0.769	14.631	0.572	13.185	-0.209
tim	142154_at	57.871	5.035	0.84	14.958	0.788	15.339	-1.089
vri	143869_at	97.001	4.238	0.838	13.268	0.839	12.564	-1.165
Pdp1	153055_at	57.909	4.827	0.442	14.644	0.332	13.625	-0.699
CG17100	142602_at	100.675	5.265	0.18	16.04	0.312	13.426	0.214
l(2)k01209	153506_at	45.847	1.426	0.14	12.57	0.005	10.32	0.007
m6	143253_at	42.084	1.238	0.051	0.315	0.067	8.208	-0.085
cbt	145534_at	39.356	2.264	0.171	15.171	0.047	12.031	-0.723
CG6767	148484_at	38.477	3.974	0.011	0.306	0.038	16.225	-0.129
sug	154772_at	37.503	2.916	0.004	18.32	0.136	14.954	-0.412
CG18317	141322_at	36.475	2.284	0.054	10.515	0.11	15.301	-0.007
l(2)08717	142212_at	36.357	6.891	0.013	13.932	0.053	19.93	0.115
CG3348	151094_at	36.312	2.785	0.168	9.497	0.126	8.989	-2.227
CG32425	149133_at	36.245	1.304	0.11	19.169	0.092	15.52	-0.043
CG31116	149709_at	34.731	1.882	0.004	7.142	0.1	13.922	0.846
zfh1	141676_at	33.67	1.185	0.013	3.313	0.045	16.717	3.281
CG13624	141772_at	33.401	2.551	0.036	5.692	0.053	13.747	0.033
CG8147	154821_at	33.087	4.497	0.343	2.515	0.152	6.38	-0.039
Thor	153432_at	32.678	3.203	0.063	2.161	0.064	4.59	0.297
Picot	142681_at	30.878	2.663	0.043	12.156	0.044	21.575	-0.013
CG12236	152818_at	30.838	1.184	0.012	10.284	0.006	9.55	0.929
HLHmbeta	151820_at	29.305	2.273	0.055	9.349	0.053	10.817	-0.127
CG15096	153069_at	29.198	5.705	0.308	20.058	0.104	20.041	0.407
CG9119	152102_at	27.949	1.447	0.325	9.451	0.049	15.228	0.164
Ugt86Dd	149663_at	27.268	2.296	0.011	3.144	0.026	19.94	0.12
l(3)02640	151808_at	26.7	1.455	0.127	21.483	0.097	22.832	-0.074
CG4797	143040_at	25.519	1.286	0.148	17.809	0.062	18.589	-0.407
CG11050	154091_at	25.349	1.493	0.343	16.819	0.16	17.876	0.309
CG8920	154008_at	23.874	1.295	0.026	3.587	0.023	13.984	0.234
ps	149557_at	23.589	1.946	0.031	18.807	0.071	13.224	-0.124
h	143186_at	23.244	1.96	0.171	13.207	0.049	19.576	0.354
CG30035	147027_at	22.382	3.729	0.005	5.233	0.043	15.785	-0.127
CG7530	155074_at	22.271	1.547	0.11	15.187	0.252	20.709	0.158
Gadd45	146727_at	21.826	1.838	0.062	18.382	0.007	10.849	-0.292
Hex-A	144761_at	20.911	1.857	0.022	1.054	0.028	17.849	0.007
CG8008	142989_at	20.759	2.336	0.291	18.802	0.396	19.976	-0.419
CG11671	149477_at	20.171	1.289	0.096	16.949	0.03	2.352	-0.151
CG6231	150208_at	19.537	3.427	0.084	12.47	0.109	14.735	1.456
CG3764	152914_at	18.885	4.06	0.095	6.313	0.03	9.895	0.262

CG10592	148228_at	18.439	2.066	0.05	11.05	0.037	1.91	-0.573
CG16826	146331_at	18.384	1.423	0.112	11.345	0.078	4.461	-3.637
Orct2	141731_at	18.369	3.592	0.113	11.005	0.12	5.776	-0.389
Mmp1	147882_at	18.246	2.115	0.053	8.35	0.192	9.644	-1.923
CG31004	150930_at	18.054	1.307	0.035	15.185	0.033	13.933	0.323
Irk3	146449_at	17.722	2.075	0.138	14.142	0.084	12.372	-0.03
CG10999	152742_at	17.133	2.485	0.006	8.543	0.093	0.818	-0.127
Cp1	141762_at	16.748	1.952	0.215	14.032	0.023	18.386	-0.079
CG3523	152126_at	15.822	1.992	0.019	13.922	0.032	12.895	0.218
CG5150	151980_at	15.767	1.364	0.077	11.539	0.098	5.488	-0.399
CG9821	141289_at	15.485	2.393	0.02	3.784	0.009	15.557	-0.05
HLHm3	143248_at	14.579	3.055	0.064	11.883	0.05	11.869	-0.163
CG13319	147122_at	13.583	1.484	0.023	1.975	0.161	4.5	-0.128
dnt	141525_at	12.68	1.395	0.084	11.331	0.112	12.558	0.152
CG1809	151956_at	12.496	1.221	0.025	18.718	0.057	2.471	-0.331
CG15044	145306_at	11.294	1.149	0.007	13.72	0.016	0.465	-0.606
AttB	147220_s_at	11.08	3.148	0.094	1.183	0.034	1.387	0.075
CG1919	148008_at	10.969	1.665	0.057	12.493	0.036	10.074	0.136
